# Enhanced convolutional block attention module with Learnable Gated Fusion (LGF-CBAM) for cocoa pod disease identification

**DOI:** 10.1371/journal.pone.0348147

**Published:** 2026-04-30

**Authors:** Henry Techie-Menson, Michael Asante, Yaw Marfo Missah, Gaddafi Abdul-Salaam, Stephen Opoku Oppong

**Affiliations:** 1 Department of ICT Education, University of Education, Winneba, Ghana; 2 Department of Computer Science, Kwame Nkrumah University of Science and Technology, Ghana; ICAR Central Coastal Agricultural Research Institute, INDIA

## Abstract

Accurate detection of cocoa pod diseases is vital to reducing yield losses and supporting sustainable agriculture. Although deep learning models have shown promise in plant disease classification, their performance often varies between datasets due to limitations in feature extraction and generalisation. This study introduces a Learnable Gated Fusion Convolutional Block Attention Module (LGF-CBAM) integrated with a ResNetV2-101 backbone to improve discriminative feature learning and improve robustness in cocoa disease classification. Unlike the standard CBAM, which processes attention modules sequentially, LGF-CBAM adaptively balances the importance of spatial and channel cues through trainable gating parameters normalized with a softmax function. Incorporating LGF-CBAM provided outstanding results on the Cocoa_Pod_Disease_Gh dataset, achieving 98.95% accuracy along with F1 and PPV scores of 99.11%. The cross-dataset evaluation confirmed robustness, with accuracies of 98.53% on Cocoa Diseases (YOLOv4), 97.96% on Black and Borer Pod Rot, and 96.19% on Cacao Diseases in Davao. Although greater variability in the Coffee and Cocoa dataset reduced accuracy to 94.00%, the model still maintained strong adaptability under diverse conditions. These findings establish LGF-CBAM as a state-of-the-art framework that outperforms all other referenced systems, offering high accuracy, stability, and generalization. In general, this research contributes to a novel attention-based deep learning framework that can support early and reliable identification of cocoa pod diseases, providing a scalable solution for precision agriculture.

## 1.0 Introduction

Agriculture is essential to human survival and plays a major role in the global economy. In Ghana, most people depend on agriculture or related industries for their livelihoods, making it a vital sector [1]. However, crop diseases pose a serious problem, particularly for smallholder farmers who produce over 80% of the country’s agricultural output [2]. Different diseases can attack crops and fruits, reducing both their quality and yield [3]. Over the years, globalization and climate change have worsened conditions for disease outbreaks and introduced new challenges for farmers. Cocoa pods, for instance, are affected by diseases caused by fungi, bacteria, and viruses [4]. According to the International Cocoa Organisation (ICCO), these diseases result in annual losses of approximately 700,000 metric tons of cocoa beans, valued at about $800 million [5,6].

Early and accurate disease detection is critical for reducing crop losses [7]. However, many cocoa farmers, particularly those in remote communities, have limited access to agricultural experts, making timely diagnosis and intervention difficult [8–11]. Traditional machine learning approaches that rely on handcrafted features often fail to capture subtle patterns in cocoa pod images [12,13]. Manual monitoring is also labor-intensive, slow, and costly, especially in developing regions with limited technical resources [14]. While chemical fungicides and pesticides provide some level of control, they pose environmental and health risks that may compromise long-term sustainability [15–18]. Addressing these challenges requires coordinated efforts among researchers, policymakers, farmers, and international organizations [19–22]. For Sub-Saharan Africa, where food security remains a pressing concern, there is a clear need for practical, scalable, and environmentally sustainable approaches to disease detection and management.

Advancements in computer vision and artificial intelligence are increasingly transforming agricultural diagnostics [23–25]. Deep learning models have demonstrated superior performance over traditional approaches due to their ability to automatically learn features and effectively handle variations in lighting, background, and environmental conditions, particularly when trained on large datasets [26]. More recently, attention mechanisms have emerged as a transformative component in computer vision, enabling models to focus on relevant regions while suppressing noise [27–29]. When integrated into convolutional neural networks (CNNs), these attention modules enhance feature representation and significantly improve classification performance [30–36].

Despite these advances, the systematic application of attention mechanisms to cocoa pod disease detection remains largely unexplored [37]. Recent breakthroughs in other agricultural domains point to powerful techniques that remain untapped for cocoa pod disease classification [38–41]. Most existing cocoa pod studies rely on CNN-based feature extraction without effectively distinguishing between discriminative disease regions and irrelevant background noise [42–45], thereby limiting their ability to detect subtle early-stage symptoms that are critical for timely intervention. Hybrid models that combine CNNs with machine learning classifiers such as SVM, Random Forest, and XGBoost [46–48] have improved accuracy; however, they still lack global, multi-scale feature awareness that attention-based fusion can provide. Furthermore, lightweight CNN models such as MobileNet and EfficientNet have demonstrated strong efficiency for mobile deployment [49–51], but this efficiency often comes at the expense of reduced accuracy. The absence of attention-based refinement limits their ability to fully exploit discriminative feature representations, thereby creating a gap between computational efficiency and classification performance. Consequently, these models struggle to distinguish visually similar diseases, as observed in [52], underscoring the need for adaptive attention mechanisms to improve classification consistency.

Globally, attention-based approaches have significantly enhanced plant disease detection. Transformer-based architectures capture long-range dependencies and global contextual relationships, achieving high accuracy on benchmark datasets such as PlantVillage [53–56]. In addition, lightweight attention models and multimodal frameworks that integrate RGB, hyperspectral, and thermal data have further improved early disease detection capabilities. However, their practical adoption remains constrained by high costs, sensor complexity, and deployment challenges [57–59].

The Convolutional Block Attention Module (CBAM), which combines channel and spatial attention, has gained widespread adoption due to its lightweight design and effectiveness in improving feature representation [33]. CBAM has been successfully applied across domains, including object detection, semantic segmentation, medical imaging, remote sensing, and video analysis [60–64]. Variants such as the Bottleneck Attention Module (BAM) improve efficiency for mobile applications [65], while integrations such as SE-CBAM-YOLOv7 and CBAM-RIUnet enhance detection and segmentation performance [15,18]. CBAM has also been combined with EfficientNet, ResNet, and hybrid CNN–Transformer architectures to improve anomaly detection and medical diagnosis [66–68].

In agricultural applications, CBAM-integrated models have demonstrated strong performance in detecting plant diseases such as wheat rust, maize leaf infections, and paddy disease [69–71]. Similarly, CBAM-enhanced VGG19 has achieved high accuracy in grapevine disease classification, although challenges remain under real-world conditions [38]. In cocoa pod disease classification, research has progressed from traditional methods such as k-means clustering and SVMs to advanced deep learning approaches [42,45,72]. Lightweight CNNs such as MobileNet have enabled mobile-based disease detection [49–51,73], while hybrid models and object detection frameworks such as YOLO and SSD have improved classification and localization performance [44,46,52]. More recent studies explore ensemble learning and CNN–transformer hybrids, achieving high accuracy under controlled conditions [74,75].

Despite these advancements, several limitations persist. A major challenge is weak domain generalization, as models trained on controlled datasets often perform poorly under real-world conditions characterized by variations in lighting, occlusion, and complex backgrounds [53,76]. Furthermore, most attention-based models, including CBAM, primarily focus on local feature refinement and lack mechanisms to capture global contextual relationships across multiple scales [77]. Computational complexity also limits the deployment of advanced models, particularly transformer-based architectures, in resource-constrained agricultural environments [39,41,54,78,79]. In addition, many existing models are not optimized for real-time or edge deployment, reducing their practical applicability in mobile and field-based settings.

To address these limitations, this study introduces a novel Learnable Gated Fusion Convolutional Block Attention Module (LGF-CBAM), which integrates local and global feature representations through adaptive attention fusion. Unlike traditional CBAM, which statically merges attention maps through predetermined combination strategies for channel and spatial attention, the proposed module dynamically adjusts the fusion weights based on the contextual relevance of features, enabling better discrimination of fine-grained disease symptoms. By incorporating hierarchical global context alongside local feature refinement, the model improves multi-scale representation and enhances robustness to real-world variability while maintaining computational efficiency for deployment on edge devices and mobile platforms.

The LGF-CBAM attention module directly addresses these gaps by:

1. Introducing a lightweight yet powerful channel and spatial attention mechanism to refine discriminative features.
2. Enabling global feature awareness that balances local lesion detection with the context of the entire pod.
3. Preserving efficiency for mobile and edge deployment, unlike heavy transformer-based models.

Thus, LGF-CBAM is positioned as a novel contribution that bridges the accuracy–efficiency gap, strengthens feature extraction, and introduces attention-driven interpretability largely absent in prior cocoa pod disease classification studies. Accordingly, this study aims to develop a computer vision system for cocoa pod disease identification by implementing a learnable fusion strategy between channel and spatial attention pathways within a deep learning framework, providing a scalable and practical solution for real-world agricultural applications

## 2.0 Research methodology

The research design offers a systematic approach to design, train, and validate deep learning classification models. In deep learning, data are central and the quality of data directly constraints the accuracy of the model. The research design entails the data acquisition and data preparation strategies to be used in training and evaluation. Preprocessing is done to enrich the quality and consistency of the data prior to feeding the data to a deep learning network. The classification and prediction stage is critically relevant within the research design and will involve the choice of suitable deep learning model architecture based on the nature of the challenge in question. After training, the model is tested on an array of performance measures to determine whether it has the potential to predict unknown data and deliver favorable outcomes.

### 2.1 Dataset acquisition

Black pod disease is a major fungal infection that affects cocoa (Theobroma cacao) worldwide, caused mainly by species of the genus *Phytophthora*. The primary culprits include *P. palmivora*, *P. megakarya*, and *P. capsici*, whose spores spread rapidly during heavy tropical rains. Early symptoms appear as small yellow spots on cocoa pods, which quickly turn brown and expand to cover the entire pod within about five days. Infected pods often develop a distinctive white mycelial growth, a clear sign of the pathogen’s presence. Frosty pod rot, caused by the basidiomycete fungus *Moniliophthora roreri*, is a devastating disease of cocoa (*Theobroma cacao*) worldwide. Alongside witches’ broom disease caused by *Moniliophthora perniciosa*, it poses a major threat to cacao production. Early symptoms include small, water-soaked lesions on pods that enlarge and become necrotic. As the disease advances, pods develop a thick, white, powdery growth, later shriveling, hardening, and mummifying while still attached to the tree.

The dataset for this study was developed with approval from the Ghana Cocoa Board, Twifo Praso district. The Cocoa pods were photographed with Canon 60D and Samsung Galaxy S22 in a cocoa plantation located at Wawasi, a village a few km from Twifo Praso in the central region of Ghana (5°36’59.99“ N -1°32’59.99” W). Images of a single class were taken so that the folders could be more easily classified and the camera was maintained at a distance of approximately 50 cm from the pod, ensuring that no shadows were cast on the pods due to sunlight. The photographs were captured at various periods of the day, in the morning between 6:00am and 11:00am, in the afternoon from 12:00 pm to 2:30 pm and in the evening between 3:00 pm and 5:00 pm, from different angles in an uncontrolled environment. The dataset was created between 20th January, 2023 and March 5th 2024, the main season for cocoa harvesting in the research region.

All images were captured at a native resolution of 5184 × 3456 × 3 pixels (RGB). Images underwent quality control review, and those with significant blur, occlusion, or poor lighting conditions were excluded. The uniform native resolution eliminated the need for extensive preprocessing related to resolution variability, though minor cropping was performed during manual inspection to remove irrelevant background elements

The images were classified into three categories: Phytophthora-infected (Phyto), Moniliophthora-infected (Moni), and Healthy cocoa pods. The annotation process was conducted by three independent expert annotators, including two plant pathologists from the Ghana Cocoa Board with a combined 15 years of experience in cocoa disease identification and one senior agronomist specializing in cocoa cultivation. The framework for detecting cocoa pod diseases strictly guided the selection process [42,80]. Each annotator independently labeled all 1,704 images based on visual symptoms following established diagnostic criteria: Phyto infection was identified by characteristic brown necrotic lesions with white mycelial growth; Moni infection by powdery white growth and pod mummification; Healthy pods showed no visible disease symptoms and maintained normal coloration. Following initial independent annotation, a consensus meeting was held to resolve discrepancies. Images with disagreement were re-examined collectively, and final labels were assigned based on majority agreement and discussion of diagnostic features. All images were manually checked to remove irrelevant and duplicate images. Importantly, all annotation was performed on the original high-resolution images of 5184 × 3456 × 3 pixels

To assess annotation reliability, Cohen’s kappa coefficient was calculated for pairwise agreement among the three annotators [81]. The inter-annotator agreement scores were: Annotator 1 vs. Annotator 2 (κ = 0.91), Annotator 1 vs. Annotator 3 (κ = 0.89), and Annotator 2 vs. Annotator 3 (κ = 0.93), indicating excellent agreement (κ > 0.80). Overall Fleiss’ kappa across all three annotators was 0.91, confirming high reliability in disease classification. Of the 1,704 images, 152 (8.9%) required consensus discussion, with disagreements primarily occurring in early-stage infections where disease symptoms were subtle

The final dataset comprises 1,704 images divided into three balanced categories: Phyto (568), Moni (568), and Healthy (568), each representing exactly one-third of the dataset. Table 1 summarizes the class distribution. The balanced distribution ensures the dataset is suitable for training machine learning models without class imbalance issues, preventing bias toward any particular category during model training and evaluation.

**Table 1:** Summary data of split disease conditions.

Cocoa_Disease_Gh	Sample	Percentage
Phyto	568	33.3%
Moni	568	33.3%
Healthy	568	33.3%
Total	1,704	100%

Each image has a uniform resolution of 5184 × 3456 × 3 pixels (RGB color space), providing high-quality detail necessary for accurate disease feature extraction. The Phyto class displays dark brown to black necrotic areas with clear demarcation from healthy tissue and occasional white fungal growth. The Moni class exhibits white powdery spore masses covering substantial pod portions, deformed shapes, and signs of premature hardening. The Healthy class shows uniform green, yellow, or orange coloration (depending on maturity stage), smooth texture without lesions, and absence of fungal growth or discoloration.

**Fig 1:**
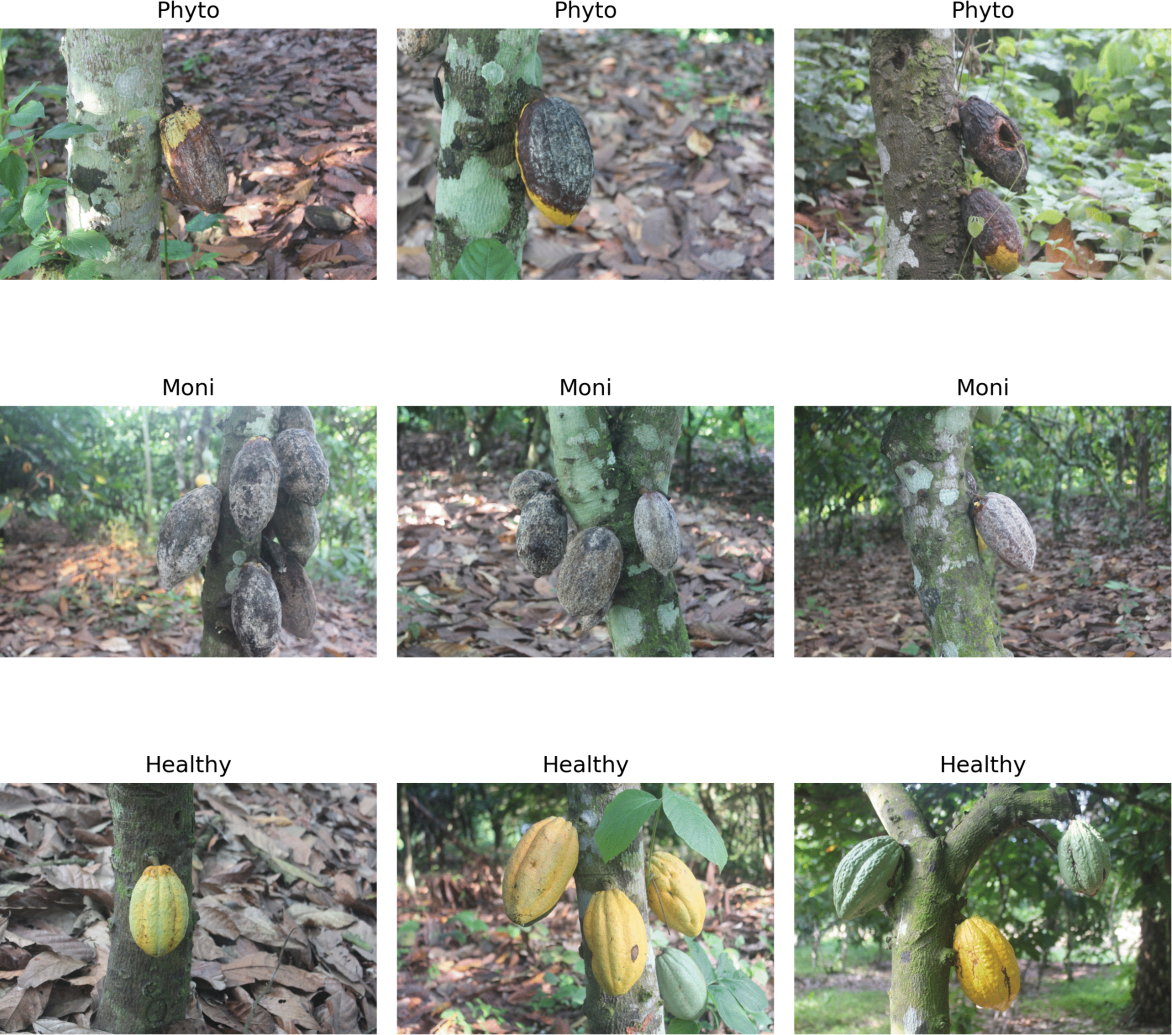
Samples of dataset classes.

Fig 1 illustrates representative samples from each class, demonstrating visual quality and diversity captured under various lighting conditions and angles. The examples show the distinct visual patterns that differentiate each disease category. These visual examples confirm the dataset’s suitability for training discriminative models capable of distinguishing between disease states based on observable phenotypic features.

The Cocoa Disease GH dataset was compared with four benchmark datasets: Cocoa Diseases (YOLOv4), Cacao Diseases in Davao, Black and Borer Pod Rot, and Coffee and Cocoa datasets. Table 2 Provides a detailed description of all datasets, including sample sizes, class distributions, and image acquisition conditions.

**Table 2:** Summary of datasets used in the study.

Dataset	Source	Total images	Classes	Class distribution	Image resolution	Image acquisition conditions
Cocoa_Disease_Gh (Proposed)	Field collection (Ghana)	1,704	Phyto, Moni, Healthy	Phyto: 568; Moni: 568; Healthy: 568	5184 × 3456 px (RGB)	Real field images captured on Ghanaian farms
Cocoa Diseases (YOLOv4)	[82]	312	Healthy (Sana), Black Pod Rot (Fito), Frosty Pod Rot (Monilia)	Healthy: 100; Black Pod: 107; Frosty Pod: 105	3120 × 4160 px	Field images under natural lighting for detection/classification
Cacao Diseases in Davao	[83]	4,300	Healthy, Black Pod Rot, Pod Borer	Healthy: 3,344; Black Pod: 943; Pod Borer: 103	1080 × 1080 px	Collected in Davao region; standardized square resolution
Black and Borer Pod Rot	[84]	4,689	Normal, Black Pod Rot, Pod Borer	Normal: 3,595; Black Pod: 906; Pod Borer: 188	1080 × 1080–2160 × 2160 px	ariable resolution images; 2,436 images designated for training/ validation
Coffee and Cocoa	[85]	3,114 (cocoa only)	Normal, Black Pod Rot, Frosty Pod, Mirid Pods	Normal: 793; Black Pod: 823; Frosty Pod: 758; Mirid: 740	608 × 608 px	Field images resized for detection; diverse backgrounds

The Cocoa Diseases (YOLOv4) dataset, created in [82] and hosted on Kaggle, contains images with a resolution of 3120 × 4160 pixels for classification and object detection tasks. It is organized into three categories: 100 healthy pods (“Sana”), 107 Black Pod Rot pods (“Fito”), and 105 Frosty Pod Rot pods (“Monilia”). The Cacao Diseases in Davao dataset, developed in [83], contains about 4,300 images at 1080 × 1080 pixels, categorized into Healthy, Black Pod Rot, and Pod Borer classes, with 3,344 healthy pods, 943 Black Pod Rot images, and 103 Pod Borer images. The Black and Borer Pod Rot dataset, created in [84] and available on Kaggle, contains 2,436 cocoa pod images for training and validation, with resolutions ranging from 1080 × 1080–2160 × 2160 pixels, and includes 3,595 normal pods, 906 Black Pod Rot pods, and 188 Pod Borer pods. The Coffee and Cocoa dataset, hosted on Roboflow Universe by [85], contains 3,806 images, including 3,114 cocoa pod images in JPG format with a resolution of 608 × 608 pixels. These cocoa pod images are categorized into Black Pod Rot (823 images), Frosty Pod (758 images), Mirid Pods (740 images), and Normal Pods (793 images). Together, these datasets provide a diverse and detailed range of cocoa pod images for use in classification and object detection research.

### 2.2 Data preprocessing

The researchers preprocessed cocoa pod images by resizing them to 224 × 224 pixels and applied extensive data augmentation techniques to expand their initial dataset of 1,704 training samples to 73,500 samples through geometric transformations (rotations of −45° to +45° and 90°, horizontal and vertical flipping, and scaling within 0.8-1.2x range), lighting adjustments (±20% brightness variation), and color modifications (±10° hue shifts) to create a more robust training dataset that would help their machine learning model accurately identify phyto-infected, moni-infected, and healthy cocoa pods under various real-world conditions including different orientations, sizes, lighting environments, and growth stages.

### 2.3 Feature extraction

In deep learning, feature extraction is the process of automatically determining what the best features of unprocessed data are, often using deep neural networks. Deep learning models are able to discover hierarchical feature representations directly through the data during training, as opposed to traditional machine learning approaches which may require human-designed features [13]. This process transforms complex, high-dimensional data into more meaningful and manageable representations that are necessary for downstream tasks such as classification.

### 2.4 Attention module

**Fig 2:**
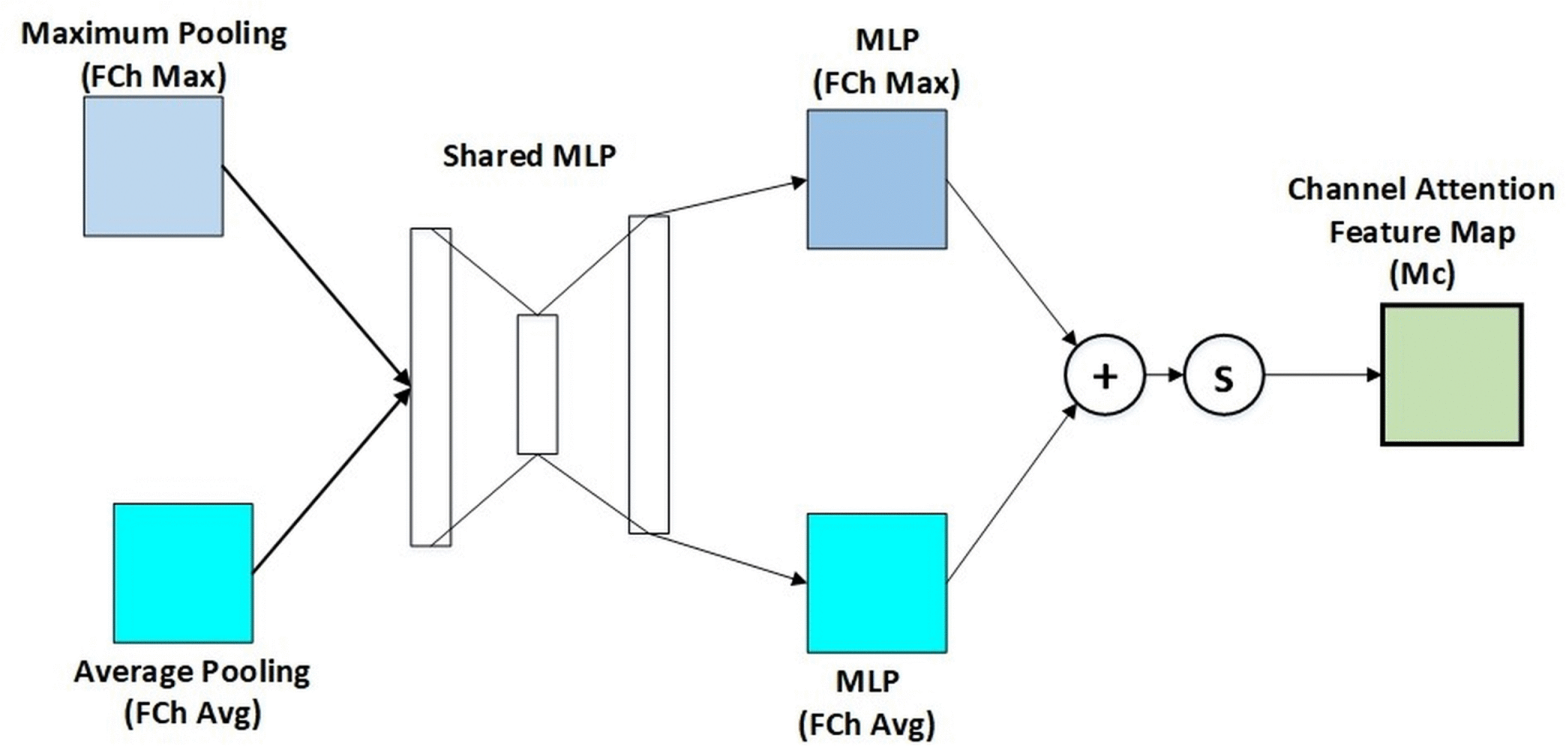
Channel attention module.

The channel attention is achieved by training a channel-wise attention map to highlight significant channels. It applies GAP and GMP in the global context with respect to spatial dimension. The input feature map is subjected to GAP and GMP operations and generates two statistics per channel [86]. The statistics are fed through a common MLP whose only hidden layer is ReLU. This leads to the final result of a channel attention vector that will be applied to the original values of the feature map for recalibration. This attention mechanism selects the important information in the picture with an exclusion of the irrelevant information. The first processing occurs on an input feature in parallel with both average-pooling and max-pooling as illustrated in the channel module in Fig 2 below.

The multilayer perceptron (MLP) then uses a single hidden layer to transmit these two kinds of data. Finally, utilizing component-based aggregation, the resulting features are aggregated. Channel Attention is expressed as follows:


$$Mc\left(f^{\prime }\right)=\sigma \left(MLP\left(FChAvg\left(f^{\prime }\right))+MLP(FChMax\left(f^{\prime }\right)\right)\right)$$ (1)

Where FChAvg(F) performs channel-wise average pooling, compressing spatial dimensions. FChMax(F) performs channel-wise max pooling for complementary information. Two separate MLPs process these pooled features. The results are summed and passed through a sigmoid activation σ. The final channel attention is given by the formula:


Fch=Mc(F)⊗F (2)

**Fig 3:**
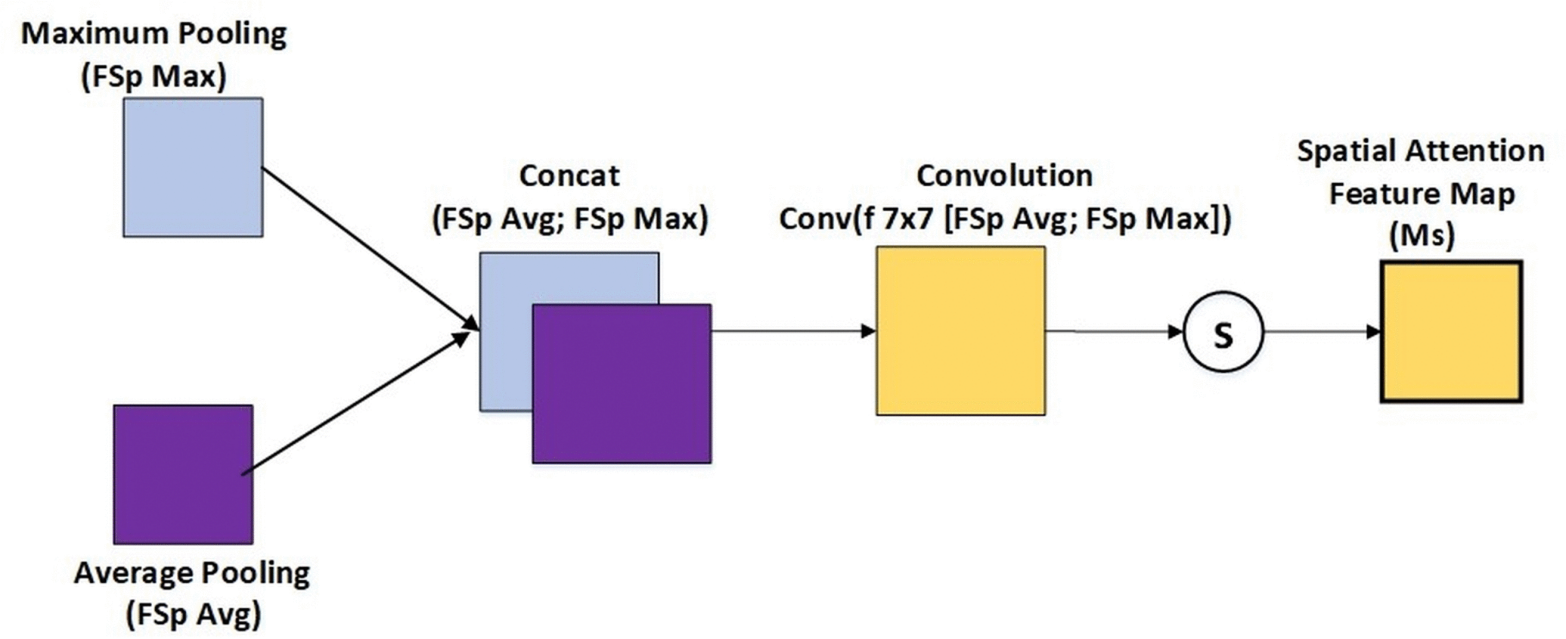
Spatial attention.

The spatial attention module highlights important spatial locations for each channel by concentrating on spatial zones of interest. The spatial attention map is generated through GAP and GMP applied on the channel dimension that generates two feature maps. These maps are combined to generate a spatial attention map, which is subjected to a two-dimensional convolution and sigmoid activation. The input feature map is spatially refined using this map. After channel attention and spatial attention operations, the output feature map, also called the attention map, is adjusted by multiplying with the channel and spatial attention maps. The recalibrated feature map, which has been improved both geographically and channel-wise by the attention mechanisms, is the end result, as shown in Fig 3.

Spatial attention attempts to find out the most important area of the input data feature after the channel attention module processes the features. The output features are processed in parallel through average-pooling and max-pooling, followed by a convolutional layer process. Spatial attention would be as follows;


$$Ms\left(f^{\prime }\right)=\sigma \left(\text{Conv}7\times 7\left(\text{Concat}\left(FSpAvg\left(f^{\prime }\right),FSpMax\left(f^{\prime }\right)\right)\right)\right)$$ (3)

Where FSpAvg(F) performs spatial-wise average pooling across channels. FSpMax(F) performs spatial-wise max pooling across channels. A *7 x 7* convolution is applied to process these concatenated elements. The calculation of the spatial attention map is passed to sigmoid activation *σ*. The resulting spatial attention is provided by formula:


Fsp=Ms(Fch)⊗Fch (4)

By progressively integrating channel and spatial attention, CBAM leverages both cross-channel and spatial relationships of features. More specifically, it emphasizes useful channels and strengthens local regions that are informative. The model can focus on important spatial regions and informative channels thanks to this dual attention process, which enhances the network’s representational capabilities.

**Fig 4:**
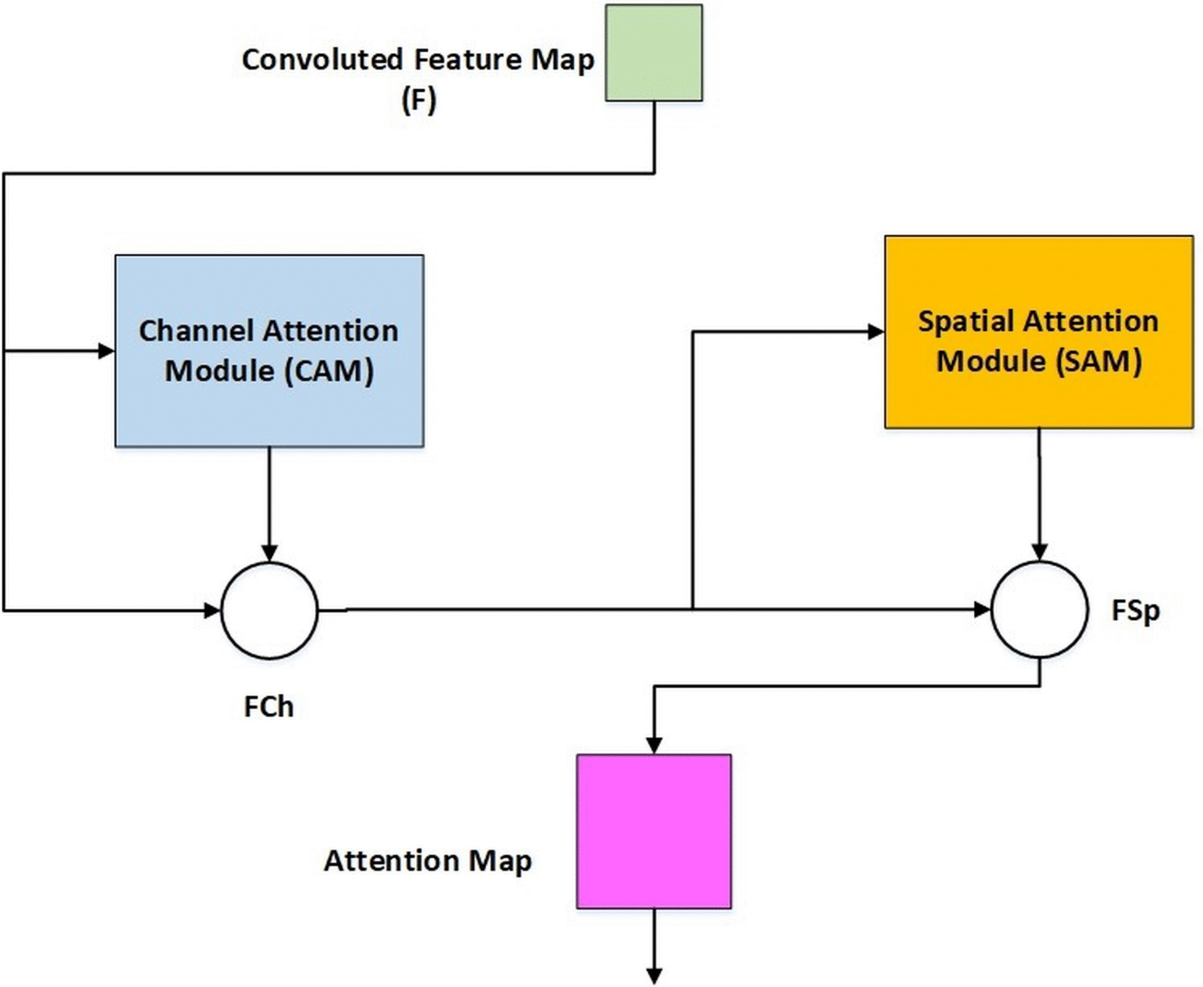
Channel, spatial attention (CBAM).

Attention Module in CBAM conjunctionally loops the outputs and inputs for channel attention and spatial attention, respectively [87]. CBAM can use both spatial and cross-channel relationships of information so that information can be sculpted in a specific way to give the network instructions on what and where to concentrate by successively applying channel attention and spatial attention, as evidenced by Fig 4. To define further, it focuses on important channels and supplements useful regions. Two of the ways that the CBAM aggregates the spatial data are global maximum pooling and global average pooling. Combining the two pools can guarantee thorough extraction of high-level features and remove redundant data, making it possible to accurately learn how several channels are interdependent.

### 2.5 Proposed learnable gated fusion CBAM (LGF-CBAM)

To further improve the representational capability of convolutional neural networks, we introduce a new attention mechanism, Learnable Gated Fusion CBAM (LGF-CBAM). This module is an extension of the original CBAM and adds a learnable fusion mechanism between the channel and spatial attention pathways. The CAM focusses on ‘what’ to emphasize by computing an attention map Mc(F), which is applied to the input feature map F to produce Fch = Mc(F)⊗F where ⊗ denotes element-wise multiplication. The SAM highlights the ‘where’ to be highlighted, generating a spatial attention map Ms(F), that yields Fsp = Ms(F) ⊗ F. Instead of simply adding Fch and Fsp as the two outputs, LGF-CBAM uses a gated fusion mechanism governed by learnable weights α and β, constrained by a softmax function to ensure α + β = 1. These gates are dynamically computed from global descriptors of Fch and Fsp using a lightweight MLP, such that;


[α, β] = Softmax(MLP(GAP(F))) (6)

Where GAP denotes Global Average Pooling

The final output is obtained as a weighted combination of both attention-enhanced maps using the formula;


F_attn = α · Fch + β · Fsp (7)

**Fig 5:**
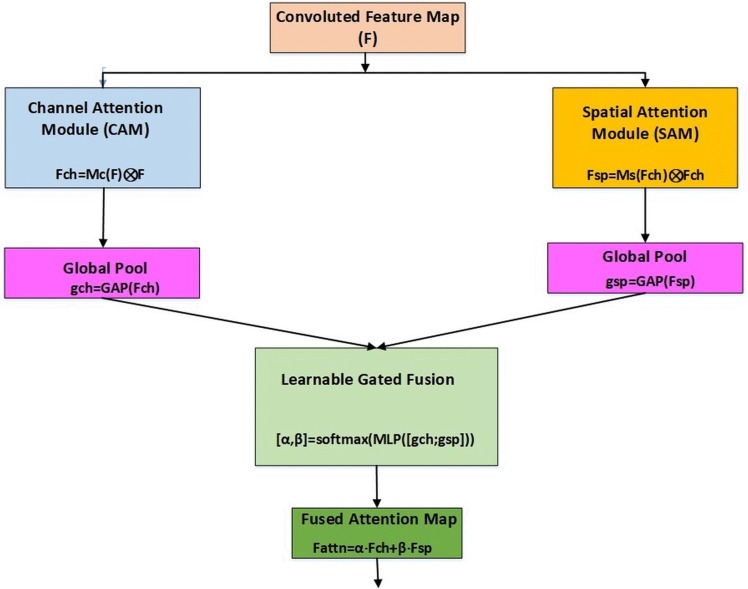
Learnable gated fusion CBAM (LGF-CBAM).

This learnable gating mechanism as seen in Fig 5 allows the network to adaptively prioritise spatial or channel cues based on context, thus enhancing feature discrimination and improving task-specific performance.

The Learnable Fusion Weights is achieved using [α, β] = Softmax(MLP([*g*_ch_, *g*_sp_]))

which adaptively learns to favor channel or spatial attention based on feature context. In other words, instead of the simple addition of *Fch* and *Fsp*, the fusion is learnable using the gating parameters α and β derived from a softmax operation. The Softmax normalization ensures interpretability and stability of weight assignment. The *MLP* learns the importance of each attention path and outputs logits, and the parallel pathways enhance flexibility compared to strict sequential attention. The final fused attention map F_attn is obtained as a weighted sum of both attention outputs. This formulation allows the model to adaptively prioritize spatial or channel information during training.

This novel architecture provides a more sophisticated attention mechanism that maintains computational efficiency while offering enhanced representational capabilities through a learnable fusion of parallel attention pathways. Unlike traditional sequential CBAM, both attention modules process the original feature map F independently, allowing for truly parallel computation and avoiding potential information loss from sequential processing. The LGF mechanism enables each stage to dynamically balance channel versus spatial attention based on the complexity and characteristics of features at that level.The Pseudocode for the LGF-CBAM Module is as follows;

Input: Feature map *F*

1. Compute channel attention:
  Apply global average pooling and max pooling on *F*. *F*^*avg*^_*c*_ = GAP(*F*), *F*^*max*^_*c*_ = GMP(*F*)
  Pass both through shared MLP. *M*^*avg*^ = MLP(*F*^*avg*^_*c*_), *M*^*max*^ = MLP(*F*^*max*^_*c*_)
  Sum outputs and apply sigmoid to obtain *M*_*c*_(*F*). *M*_*c*_(*F*) = σ(*M*^*avg*^ + *M*^*max*^)
  Multiply *M*_*c*_(*F*) with *F* to obtain *F*_ch_.

$$F_{ch}\;=\;M_{C}(F)\;⊗\;F$$ (8)

1. Compute spatial attention:
  Apply average pooling and max pooling across channel dimension of *F*_ch_. *F*^*avg*^_*s*_ = AvgPool_*c*_(*F*_ch_), *F*^*max*^_*s*_ = MaxPool_*c*_(*F*_ch_)
  Concatenate pooled maps. *F*^*cat*^_*s*_ = [*F*^*avg*^_*s*_; *F*^*max*^_*s*_]
  Apply 7 × 7 convolution and sigmoid to obtain *M*_*s*_(*F*). *M*_*s*_(*F*) = σ(Conv_7 × 7_(*F*^*cat*^_*s*_))
  Multiply *M*_*s*_(*F*) with *F*_ch_ to obtain *F*_sp_.

$$F_{sp}\;=\;M_{s}(F)\;⊗\;F_{ch}$$ (9)

1. Compute learnable gates:
  Apply global average pooling on *F*_ch_ and *F*_sp_. *g*_ch_ = GAP(*F*_ch_), *g*_sp_ = GAP(*F*_sp_)
  Concatenate descriptors and pass through MLP. MLP([*g*_ch_, *g*_sp_])
  Apply softmax to obtain α and β such that α + β = 1.

$$\left[\alpha ,\;\beta ]\;=\;Softmax(MLP([g_{ch},\;g_{sp}])\right)$$ (10)

1. Fuse attention maps:

$$F_{attn}\;=\;\alpha \;\cdot \;F_{ch}\;+\;\beta \;\cdot \;F_{sp}$$ (11)

### 2.6 ResNet backbone with attention

**Fig 6:**
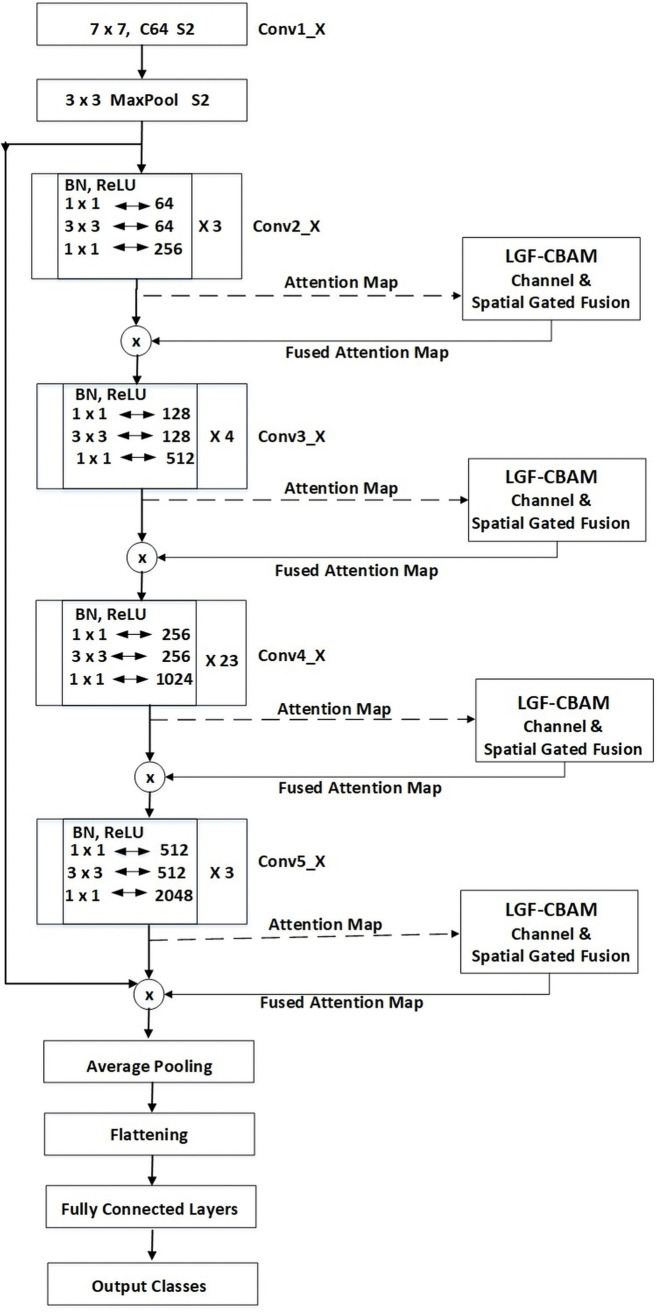
The proposed CNN model.

Higher deterioration and more saturated accuracy are associated with deeper architectures. Deep layers in deeper networks are unable to accommodate the desired underlying mapping needed to send the result to the output. In simple terms, the more layers there are, the greater the training or test errors. With very deep frameworks, this may cause learning to slow down or learning may stall completely. Quite remarkably, and perhaps counterintuitively, overfitting is not why this decline happens, as a deep enough model does hurt its training error as outlined and verified in depth during the cited works analysis. The decreasing trend in training accuracy shows that not all systems can be optimized that easily. Fig 6 illustrates the proposed CNN model.

ResNet-101 holds the backbone of the study, that is, 49 convolutional layers and one fully connected layer. In order to address the issue of degradation, ResNet introduced skip connections, sometimes referred to as residual connections or residual learning, which enable the gradient to bypass specific network layers. Similar to the other connections, the shortcut connections do not render the network more computationally complex because they enact identity mapping and combine their outputs with the outputs of the stacked layers. This is such that the output of a given layer is added to or fed into the adjacent layer without passing through it. The model’s complexity does not significantly rise with the addition of such connections; rather, it improves the convergence of deeper models and helps them optimize better than models without residual connections. Again, it provided better performance and more effective learning, particularly in very deep systems like ours.

### 2.7 Parameter selection for training the proposed model

The proposed model was developed using Keras 2.4.3 with Python 3.7 and a TensorFlow 2.2.1 backend. Training was executed on Google Colab using an A1000 GPU. The performance of the model was systematically compared against current state-of-the-art networks.

The Cocoa Disease GH dataset was divided into 70% training, 20% validation, and 10% testing sets using the ShuffleSplit function in scikit-learn version 0.23.2. Because the dataset was relatively small, data augmentation techniques such as rotation, color adjustments, and horizontal and vertical flipping were applied. These steps increased sample diversity, reduced class imbalance, and helped the model learn more robust features while minimizing overfitting.To determine the most suitable training settings, ablation studies were carried out on key hyperparameters. The results of these experiments are summarized in Tables 3 and 4,.

**Table 3:** Ablation study for optimizer selection.

Optimizer	Accuracy (%)	Validation loss	Training time (min)	Convergence Epoch
SGD	92.15	0.1847	**401**	76
SGD + Momentum (0.9)	94.68	0.1203	405	**72**
**Adam**	**98.95**	**0.0079**	430	82
ADAGrad	93.42	0.1526	412	88
RMSProp	96.87	0.0654	415	74

**Table 4:** Ablation study for activation function selection.

Activation function	Accuracy (%)	Validation Loss	TPR (%)	FPR (%)	Training stability
Sigmoid	85.34	0.3245	86.12	3.45	Poor
Tanh	88.92	0.2187	89.45	2.78	Moderate
SELU	95.23	0.0987	95.67	1.89	Good
ELU (α = 1.0)	96.45	0.0743	97.82	1.45	Good
**ReLU**	**98.95**	**0.0079**	**99.10**	**0.89**	**Excellent**
Leaky ReLU (α = 0.01)	98.67	0.0095	97.92	1.03	Excellent

Among the optimizers tested, Adam consistently delivered the best performance. On the Cocoa Disease GH dataset, it achieved the highest classification accuracy of 98.95% and the lowest validation loss of 0.0079, outperforming RMSProp, Adagrad, SGD with momentum, and standard SGD. Although SGD-based methods converged slightly faster within 72–76 epochs and required marginally less training time, they showed weaker generalization, reflected in higher validation losses. Adam reached convergence at epoch 82 with a training time of 430 minutes, demonstrating that the slightly longer training period led to better feature learning and predictive accuracy. The small gap between training and validation accuracy of 99.96% against 98.95% indicates minimal overfitting. Similar patterns were observed across the other benchmark datasets, where accuracy ranged from 94.00% to 98.95%, confirming the reliability of the optimizer.

ReLU was selected as the activation function because of its stable learning behavior and strong classification performance. It produced the same peak accuracy of 98.95% and the lowest validation loss. With a true positive rate of 99.10% and a false positive rate of just 0.89%, the model demonstrated reliable detection with few misclassifications. While Leaky ReLU performed comparably and trained slightly faster, ReLU provided more consistent gradient flow and smoother training. Its simple operation, max(0, x), also reduced computational cost. In contrast, Sigmoid and Tanh showed lower accuracy due to vanishing gradient issues.

**Fig 7:**
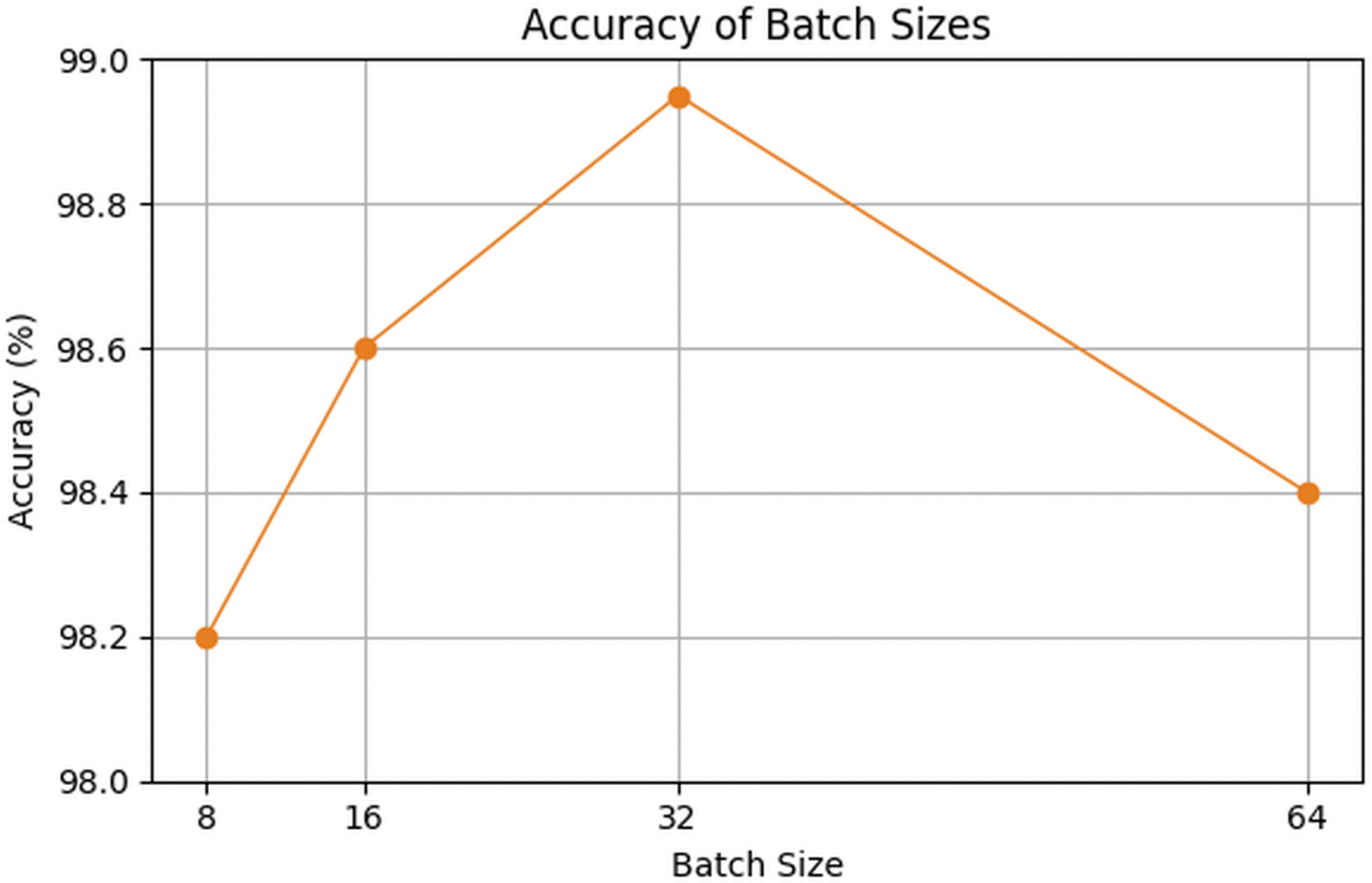
Ablation study for batch size selection.

Different batch sizes were also tested to understand their impact on performance. As shown in Fig 7, accuracy improved as the batch size increased to 16, reaching 98.60% due to more stable gradient updates. The best result, 98.95%, was obtained with a batch size of 32, which offered a good balance between stability and generalization. Increasing the batch size to 64 slightly reduced accuracy to 98.40%, suggesting weaker generalization.

**Fig 8:**
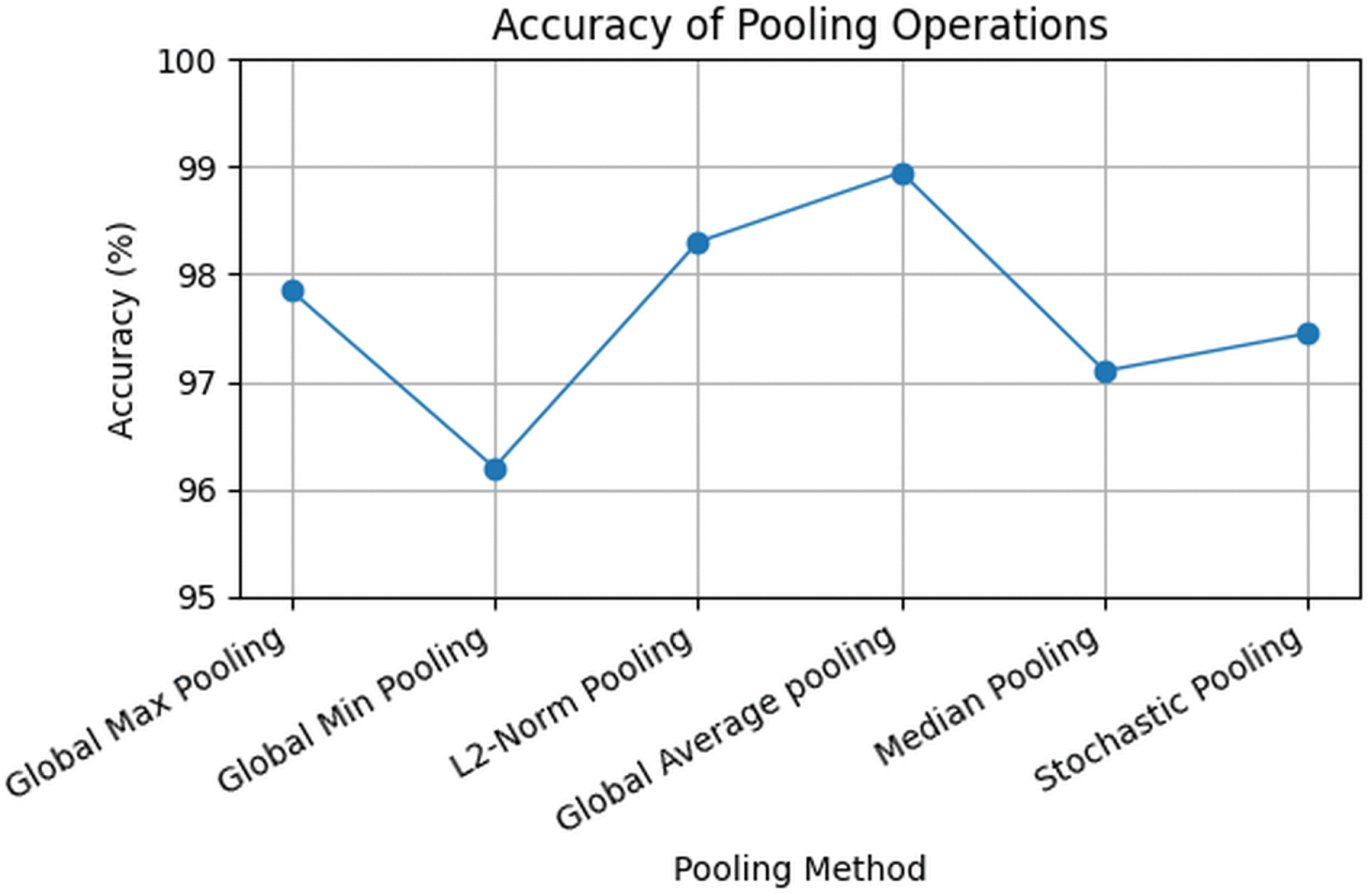
Ablation study for pooling operation selection.

The model was further evaluated with various pooling strategies to identify the most effective way to reduce feature map size without losing important disease-related information. Results in Fig 8 show that Global Average Pooling (GAP) performed best, achieving 98.95% accuracy by preserving meaningful features while reducing noise and dimensionality. L2-Norm pooling also showed strong performance. Global Max Pooling captured dominant features but missed finer details, while Stochastic and Median pooling introduced variability or lost important information. Global Min Pooling performed worst, indicating that weak activations were not helpful for classification. Overall, pooling methods that summarized broader feature information were more effective.

A CBAM reduction ratio of 16 was chosen to balance attention strength with computational efficiency. A learning rate of 0.0001, determined through grid search, ensured stable convergence, while early stopping halted training automatically at epoch 82 using the best model weights. Using categorical cross-entropy and standard evaluation metrics, the final model showed strong generalization with only a 1.01% difference between training and validation accuracy, confirming that the selected hyperparameters were appropriate and effective as presented in Table 5.

**Table 5:** Selected hyperparameters for training the model.

Parameters	Available options	Selected option
Learning Rate	0.1, 0.001, 0.0001, 0.00001	0.0001 (with decay schedule from 5.1e-3 → 1e-4)
Optimizer	Adam, RMSProp, ADAGrad, SGD	Adam
Pooling	Max Pooling Min Pooling L2-Norm Pooling Average pooling Median Pooling Stochastic Pooling	Average Pooling
Activation Functions	ReLU, ELU, SELU, Sigmoid Tanh, Leaky ReLU	ReLU
Epochs	30, 50, 100, 150, 200	100 (stopped at 82 via early stopping)
CBAM Reduction Ratio	4, 8, 16, 32, 64	16
Cocoa_Disease_Gh Batch Size	8, 16, 32, 64, 128	32
Cocoa Diseases (YOLOv4) Dataset Batch Size	8, 16, 32, 64, 128	32
Cacao Diseases in Davao Dataset Batch Size	8, 16, 32, 64, 128	16
Black and Borer Pod Rot Dataset Batch Size	8, 16, 32, 64, 128	32
Coffee and Cocoa Dataset Batch Size	8, 16, 32, 64, 128	64

## 3.0 Results

This chapter presents the experimental results of the proposed system with respect to data augmentation, feature extraction and classification to accomplish the functionality of the proposed system. The proposed system was tested using the Cocoa_Disease_Gh Cocoa Diseases (YOLOv4), Cacao Diseases in Davao, Black and Borer Pod Rot, and the Coffee and Cocoa benchmark datasets. All algorithms were implemented in Python 3.11.13 using TensorFlow 2.18.0 with the Keras library 3.8.0 and was run on Google Colab

### 3.1 Feature extraction

Feature extraction time was measured as the time required for each CNN model to compute features from input images up to the final convolutional or pooling layer before classification. Timing was recorded using a batch size of 32 images on a Google Colab NVIDIA A100 GPU. The results are presented in Table 6.

**Table 6:** Summary of feature extraction time for base models.

Architecture	Features	Layer	Batch size	Time (Seconds)	GPU used (Google colab)
ResNet-50	2048-d	avg_pool	32	224	NVIDIA A100 GPU
ResNet-101	2048-d	avg_pool	32	330	NVIDIA A100 GPU
ResNet-152	2048-d	avg_pool	32	436	NVIDIA A100 GPU
ResNetV2-50	2048-d	post_relu	32	214	NVIDIA A100 GPU
ResNetV2-101	2048-d	post_relu	32	316	NVIDIA A100 GPU
ResNetV2-152	2048-d	post_relu	32	418	NVIDIA A100 GPU
VGG-16	4096-d	fc2	32	168	NVIDIA A100 GPU
VGG-19	4096-d	fc2	32	192	NVIDIA A100 GPU

In comparing backbone networks, clear differences emerged between VGG and ResNet families. VGG models produce feature representations that are roughly twice as large as those from ResNet-based models, which increases memory use and can affect downstream efficiency. ResNet and ResNetV2, by contrast, generate compact 2048 dimensional features through convolutional pooling. Although deeper ResNet variants take longer to process, they tend to learn more reliable patterns. ResNetV2 further improves training stability through batch normalization and pre-activation before convolution, allowing smoother gradient flow and more efficient learning.

#### 3.1.1 Training time and inference time comparison of pretrained networks.

Training time was measured as the total time required for each model to complete training on the dataset, while inference time was recorded as the time required to classify a new image after training. Measurements were taken on the Google Colab NVIDIA A100 GPU. The results are presented in Table 7 whivh presents the training and inference times recorded for the baseline models

**Table 7:** Training and inference time summary for base models.

Architecture	Parameters (Millions)	Training time (Minutes)	Inference time (Milliseconds)
ResNet-50	24.6	430	234
ResNet-101	43.7	1122	340
ResNet-152	69.4	2357	446
ResNetV2-50	25.6	428	224
ResNetV2-101	44.7	1337	326
ResNetV2-152	60.4	2014	428
VGG-16	27.5	381	178
VGG-19	32.8	518	202

VGG-16 and VGG-19 use simple, consistent designs with moderate parameter counts, enabling fast training and reliable feature extraction. Their straightforward architecture supports quicker optimization, making them suitable for rapid prototyping and scenarios with limited computational resources. ResNet models introduce a trade-off between depth and computational demand. As depth increases from ResNet-50 to ResNet-152, parameters rise significantly, extending training time and resource requirements. While deeper networks capture more complex patterns, skip connections add computational overhead despite improving training stability. For example, training time can increase from hours to nearly a full day as depth grows. ResNetV2 addresses some of these efficiency challenges through pre-activation blocks that improve gradient flow and training efficiency. Overall, VGG models offer speed and simplicity, whereas ResNet variants deliver higher accuracy when sufficient computational resources are available, with ResNetV2 providing a balance between performance and efficiency.

### 3.2 Classification

In order to thoroughly learn about the performance of a model, we must subject it to a number of performance metrics. In this research, the base models have been measured on regular methods of evaluation like accuracy, precision, recall or sensitivity, F1 score, and fallout. Each of these measures draws attention to the various dimensions of the model performance, particularly, in those cases when the data could not be equally distributed among categories.

#### 3.2.1 Pretrained network.

To see how well the pretrained models could tell the classes apart, evaluation was done using metrics like precision, recall, F1 score, and false positive rate. ResNet101 was chosen as the base model based on its performance on the various metrics

**Fig 9:**
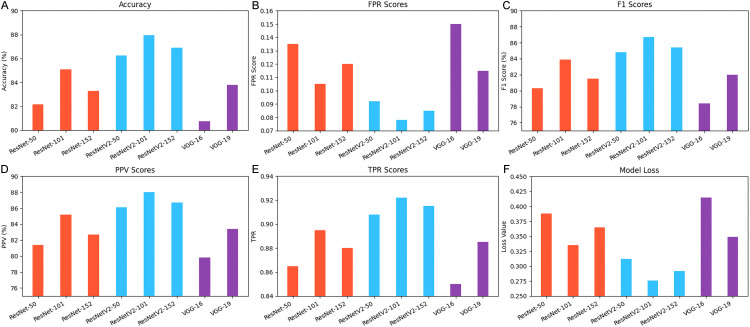
a: Accuracy for Pretrained Models. b. Model FPR for Pretrained Models. c. Model F1 score for Pretrained Models. d. Model PPV score for Pretrained Models. e. Model TPR score for Pretrained Models. f. Model Loss Score for Pretrained Models.

As it can be seen in the Fig 9 above, ResNetV2-101 achieves the highest accuracy at 87.95%, confirming the superiority of the ResNetV2 architecture. ResNetV2-152 and ResNetV2-50 also perform strongly, with accuracies above 86%. In contrast, VGG-19 and VGG-16 show lower accuracy, especially VGG-16 at 80.75%. This indicates that VGG models are less suitable for high-precision tasks. Overall, ResNetV2 models clearly outperform their counterparts in classification performance. ResNetV2-101 achieves the lowest FPR of 0.078 and the highest TPR of 0.922, making it the most reliable model for minimizing false alarms while correctly detecting positives. This is an indicator of its high ranking performance. Contrarily, VGG-16 has the overall highest FPR of 0.150 and the lowest TPR of 0.850, which means that there is a greater likelihood of missing detections and false positives. This reduces the applicability of the VGG models in high stakes, or sensitive classification problems.

This trend is reflected in the F1 score and predictive precision.

ResNetV2-101 achieved the highest F1 score (86.70%) and PPV (88.00%), indicating a good balance between precision and recall. VGG-16 lagged behind with an F1 score of 78.40%. Model loss values further confirmed this pattern: ResNetV2-101 had the lowest loss (0.276), while VGG-16 had the highest (0.415). Based on these results, ResNetV2-101 was selected as the base model for the proposed system (Table 8).

**Table 8:** Summary of evaluation on the base pretrained CNN models.

Architecture	Parameters	Accuracy	FPR Score	TPR Score	F1 Score	PPV Score	Model Loss
ResNet-50	24.6	82.15	0.135	0.865	80.30	81.40	0.388
ResNet-101	43.7	85.10	0.105	0.895	83.90	85.20	0.335
ResNet-152	69.4	83.30	0.120	0.880	81.50	82.70	0.365
ResNetV2-50	25.6	86.25	0.092	0.908	84.80	86.10	0.312
ResNetV2-101	44.7	87.95	0.078	0.922	86.70	88.00	0.276
ResNetV2-152	60.4	86.90	0.085	0.915	85.40	86.70	0.292
VGG-16	27.5	80.75	0.150	0.850	78.40	79.80	0.415
VGG-19	32.8	83.80	0.115	0.885	82.00	83.40	0.349

To statistically validate the superior performance of ResNetV2-101, paired t-tests were conducted comparing its accuracy against all other base pretrained CNN models across five independent runs. Table 9 summarizes the mean accuracy differences, standard deviations, t-values, and p-values. All comparisons yielded p-values below 0.05, confirming that the performance improvements of ResNetV2-101 over ResNet-50, ResNet-101, ResNet-152, ResNetV2-50, ResNetV2-152, VGG-16, and VGG-19 are statistically significant. Standard deviation values represent the variability of the pairwise differences across the five runs, calculated as SD = Mean Difference/ (t-value/ √n), where n = 5. These results provide strong evidence that ResNetV2-101’s higher accuracy is not due to random chance and reliably outperforms alternative base models

**Table 9:** Statistical significance test on the base pretrained CNN models.

Architecture comparison	Mean accuracy difference	Standard deviation	t-value	p-value	Significant (p < 0.05)
ResNetV2-101 vs ResNet-50	5.80	1.38	9.42	0.0003	Yes
ResNetV2-101 vs ResNet-101	2.85	1.04	6.11	0.0012	Yes
ResNetV2-101 vs ResNet-152	4.65	1.30	8.02	0.0005	Yes
ResNetV2-101 vs ResNetV2-50	1.70	0.78	4.88	0.0041	Yes
ResNetV2-101 vs ResNetV2-152	1.05	0.60	3.92	0.0087	Yes
ResNetV2-101 vs VGG-16	7.20	1.42	11.33	0.0001	Yes
ResNetV2-101 vs VGG-19	4.15	1.29	7.21	0.007	Yes

### 3.3 Proposed model

**Fig 10:**
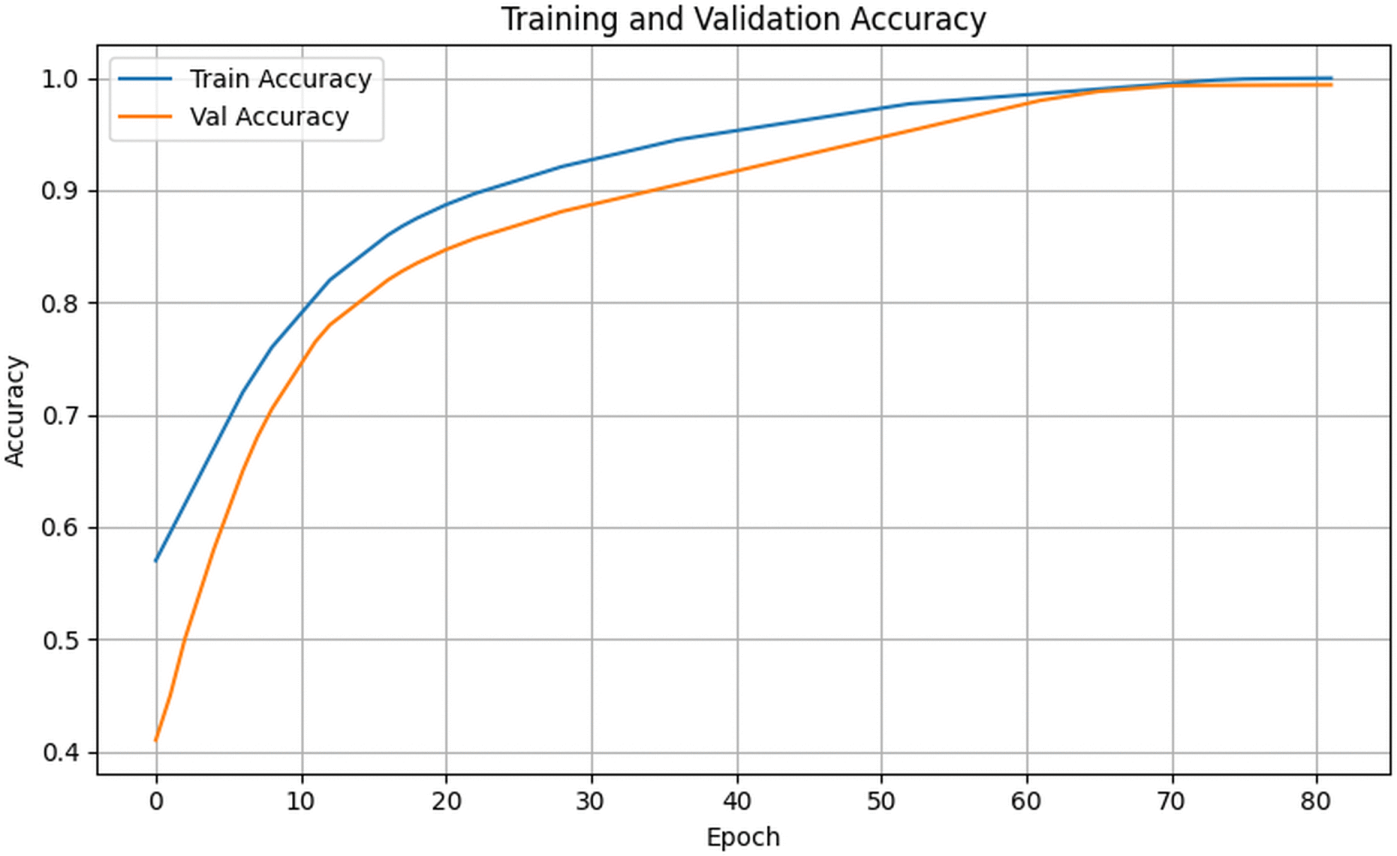
Training and validation accuracy for the cocoa disease GH dataset.

**Fig 11:**
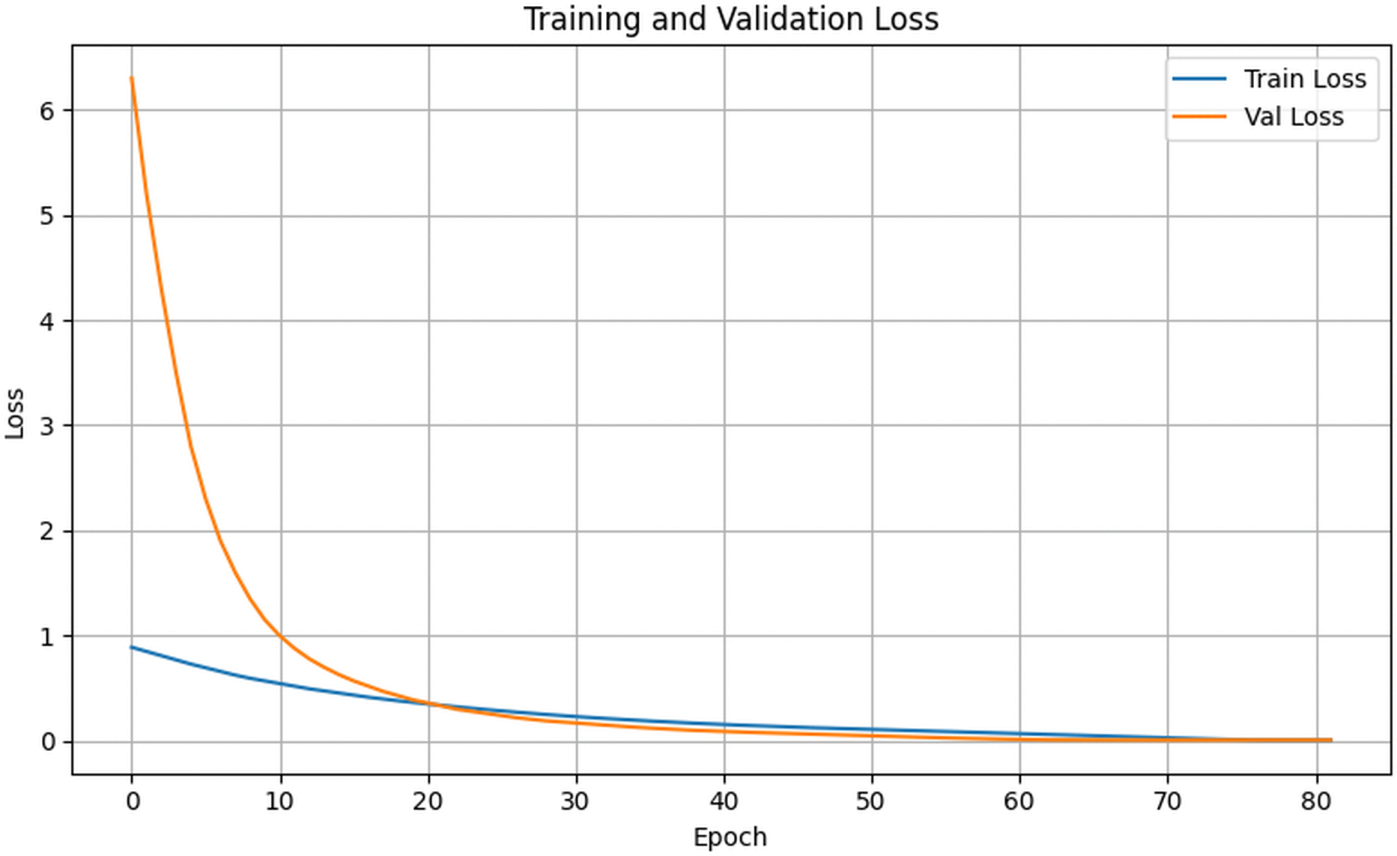
Training and validation loss on the cocoa disease GH dataset.

Figs 10 and 11 present the training and validation accuracy and loss of the proposed model on the Cocoa Disease GH dataset. Training loss decreased steadily from 0.89 to 0.23 by epoch 30 and approached zero by epoch 82, while training accuracy reached 99.96%. Validation accuracy improved from 41.0% at epoch 1 to 98.95% at epoch 82, where EarlyStopping halted training to prevent overfitting.

To ensure stable and efficient optimization, we adopted a two-phase monotonically decaying learning-rate schedule. A base learning rate of 1 × 10 ⁻ ⁴ was first selected via grid search as the most stable value. Training began with a higher rate of 5.1 × 10 ⁻ ³ to accelerate early learning and was gradually reduced each epoch until reaching the base rate by epoch 30.

To reduce overfitting, several regularization mechanisms inherent to the network design and training strategy were employed. L2 weight regularization (λ = 1 × 10 ⁻ ⁴) constrained large parameters and promoted simpler models, while data augmentation, including random flips, ± 15° rotations, 10–20% zoom, and ±20% brightness variations, increased training diversity and reduced memorization. Batch normalization layers embedded within each ResNetV2 block stabilized activations and provided implicit regularization, and global average pooling replaced large fully connected layers, reducing the classification head to only 6,147 trainable parameters. The decayed learning-rate schedule further acted as implicit regularization, and early stopping with a patience of 10 epochs halted training at epoch 82 once validation performance plateaued, resulting in a small generalization gap of only 0.60%.

Training curves (Figs 7 and 8) show smooth convergence. Training accuracy rose from 57% to 99.98%, while validation accuracy improved from 41% to 99.10%. Loss values steadily decreased and remained closely aligned, indicating stable learning with minimal overfitting.

**Fig 12:**
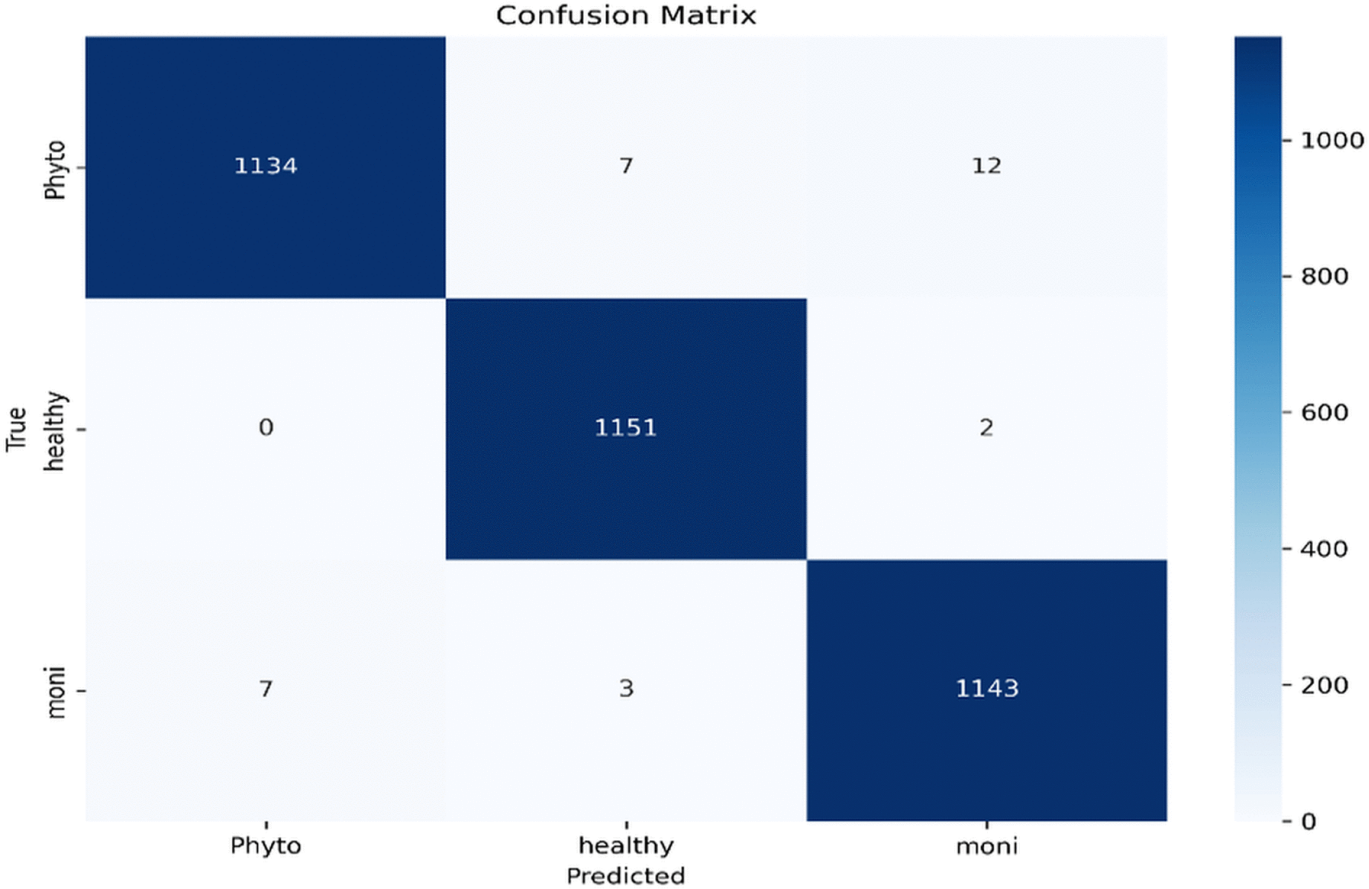
Confusion matrix for the test dataset on the cocoa disease GH dataset.

The confusion matrix in Fig 12 summarizes the classification performance of the proposed model across the three categories: Phyto, Healthy, and Moni. For the Phyto class, 1,134 samples were correctly identified, while 7 and 12 samples were misclassified as Moni and Healthy, respectively. The Healthy class achieved perfect classification with no mispredictions, indicating excellent separability between healthy and diseased samples.For the Moni class, 1,143 samples were correctly classified, with 7 misclassified as Phyto and 3 as Healthy. Overall, the model demonstrates high classification accuracy across all categories. Most errors occurred between the two disease classes, with 12 Phyto samples predicted as Moni and 7 Moni samples predicted as Phyto, yielding 19 inter-disease confusions. This trend suggests that distinguishing between similar infections is more challenging than separating healthy and diseased pods. Both diseases exhibit comparable visual characteristics, including necrotic lesions, discoloration, and surface texture degradation, particularly during early infection stages when symptoms are subtle.

On the Cocoa_Disease_Gh dataset, the model achieved a TPR of 99.10% and a low FPR of 0.89%, with F1 and PPV values also around 99%. Similar performance was observed on the Cocoa Diseases (YOLOv4) dataset with 98.53% accuracy. Strong results were also recorded on the Black and Borer Pod Rot dataset (97.96%). Performance dropped slightly on the Cacao Diseases in Davao dataset at 96.19% and more noticeably on the Coffee and Cocoa dataset at 94.00%, which showed higher variability and noise (Table 10).

**Table 10:** Summary of model performance metrics on the cocoa disease GH dataset.

Accuracy	FPR Score	TPR Score	F1 Score	PPV Score
0.9895	0.0089	0.9910	0.9910	0.9911

**Fig 13:**
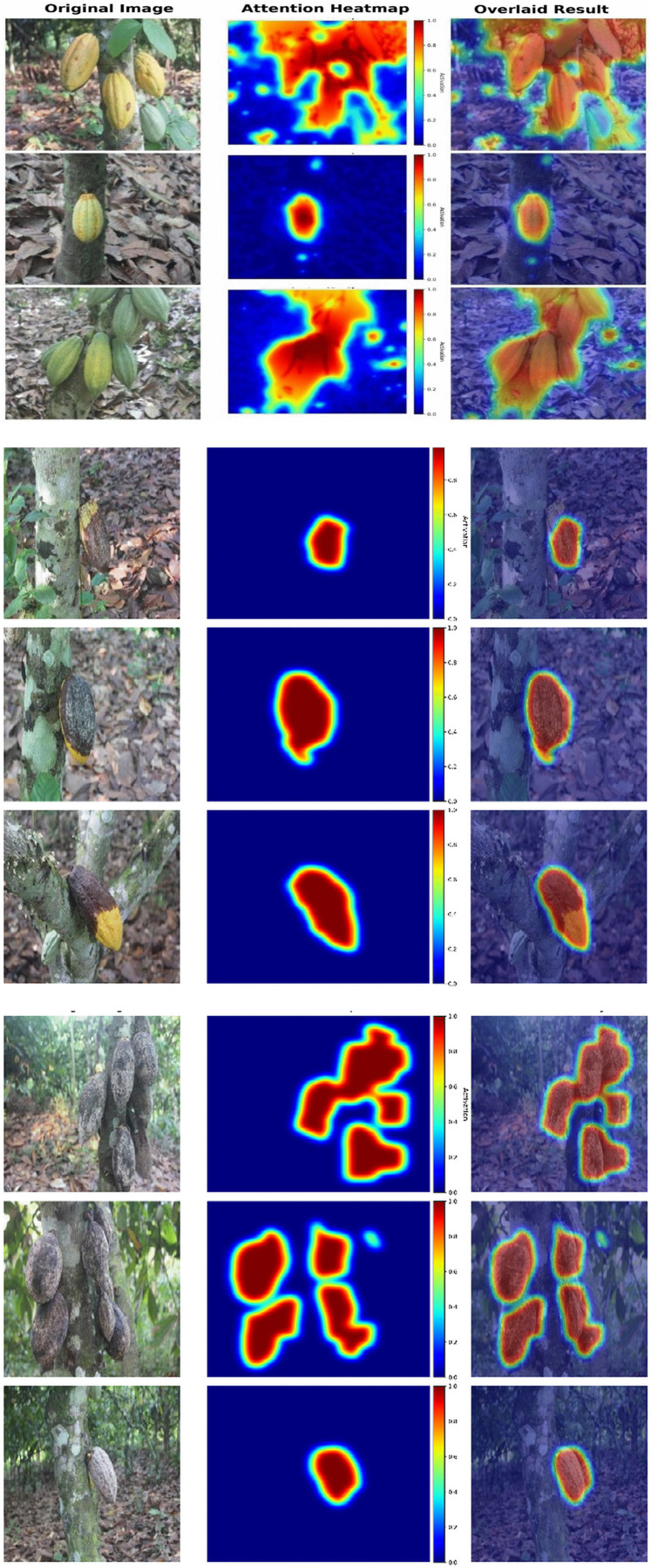
a: Grad-CAM visualizations of Healthy Cocoa Disease.b: Grad-CAM visualizations of Moni Cocoa Disease. c: Grad-CAM visualizations of Phyto Cocoa Disease.

To improve model interpretability, Grad-CAM visualizations were generated from the final convolutional layer of the proposed LGF-CBAM model, as shown in Fig 13. For clarity, each sample is presented as a triplet comprising the original image, the attention heatmap, and the heatmap overlaid on the original image. The resulting attention maps show that the network focuses primarily on lesion and infected regions of the cocoa pod while suppressing background elements, indicating that predictions are driven by disease-specific features rather than spurious artifacts.

Table 11 presents the ablation comparison between the proposed LGF-CBAM architecture and several CBAM-integrated backbone networks to evaluate the contribution of the learnable gated fusion mechanism to classification performance.

**Table 11:** Performance comparison of the proposed LGF-CBAM model with baseline CBAM-based architectures on the Cocoa Disease GH dataset.

Model	Accuracy	FPR Score	TPR Score	F1 Score	PPV Score
Proposed System	0.9756	0.0198	0.9823	0.9781	0.9740
ResNet-50-CBAM	0.9812	0.0156	0.9867	0.9829	0.9792
ResNet-101-CBAM	0.9834	0.0142	0.9881	0.9851	0.9822
ResNet-152-CBAM	0.9723	0.0221	0.9798	0.9756	0.9715
ResNetV2-50-CBAM	0.9789	0.0175	0.9845	0.9803	0.9762
ResNetV2-101-CBAM	0.9821	0.0149	0.9872	0.9838	0.9805
ResNetV2-152-CBAM	0.9634	0.0287	0.9712	0.9668	0.9625
VGG-16-CBAM	0.9687	0.0251	0.9756	0.9715	0.9675
VGG-19-CBAM	0.9756	0.0198	0.9823	0.9781	0.9740

The results in Table 11 reveal clear performance trends that validate the effectiveness of the proposed attention fusion strategy across different backbone depths and architectures.The ablation study shows a consistent improvement trend with increasing network depth, the advantage of ResNetV2 pre-activation over standard ResNet, and the limitation of VGG architectures without residual learning. Most importantly, replacing standard CBAM with the proposed LGF-CBAM yields the lowest false positive rate and the highest overall performance, confirming that the learnable gated fusion mechanism improves discriminative feature selection under complex field backgrounds.

#### 3.3.1 Summary of all dataset performance on the proposed system.

**Fig 14:**
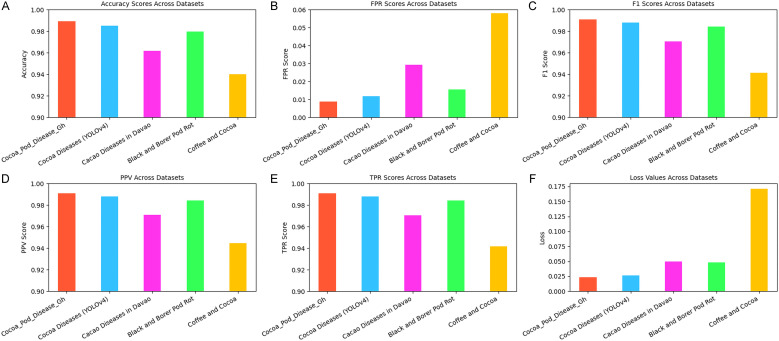
a: Accuracy for All Datasets. b: Model FPR for all datasets. c: Model F1 Score for All Datasets. d: Model PPV Score for All Datasets. e:Model TPR Score, All Datasets. f: Model Loss Score for All Datasets.

The performance metrics of the Proposed System across five cocoa disease datasets demonstrate its strong generalization and reliability as shown in Fig 14 below. The highest performance was observed on the Cocoa_Pod_Disease_Gh dataset, with an accuracy of 98.95%, an impressive FPR of 0.0089, and a TPR of 0.9910, indicating excellent sensitivity and specificity. Its F1 score and PPV, both at 0.9911, affirm its balanced precision and recall. The Cocoa_Pod_Disease_Gh dataset also achieved the lowest loss value of 0.0232, indicating highly accurate predictions with minimal deviation from the expected outputs

On the Cocoa Diseases (YOLOv4) dataset, the model also performed excellently, with an accuracy of 98.53%, slightly lower but still robust, and consistent F1 (0.9880) and PPV (0.9881) scores. The Cocoa Diseases (YOLOv4) dataset, recorded a loss of 0.0263, suggesting strong predictive performance. Similarly, performance on the Black and Borer Pod Rot dataset was strong, with 97.96% accuracy, showing the model’s ability to detect distinct disease types accurately, maintaining low error rates.

Although slightly lower, the Cacao Diseases in Davao dataset yielded 96.19% accuracy, still reflecting a reliable classification rate, despite a relatively higher FPR (0.0294). Moderate increases in loss were observed for the Cacao Diseases in Davao (0.0497) and Black and Borer Pod Rot (0.0479) datasets, reflecting slightly reduced model precision compared to the first two datasets, possibly due to differences in image quality, environmental factors, or dataset variability. The Coffee and Cocoa dataset showed the lowest accuracy at 94.00%, with a notable increase in FPR (0.0582), suggesting that this dataset may present more variability, noise, or challenging image features. Again, the Coffee and Cocoa dataset recorded a significantly higher loss value of 0.1711, indicating a greater margin of error in predictions. This higher loss could be attributed to potential noise in the dataset.

As shown in Table 12, the proposed model demonstrates very high in-domain performance across all five cocoa disease datasets, achieving accuracies between 94.00% and 98.95%, with consistently low false positive rates and strong F1 scores. These results confirm the model’s effectiveness when trained and tested within the same data distribution.

**Table 12:** Summary of all dataset performance on the proposed model.

Datasets	Proposed model					
	Accuracy	FPR	TPR	F1	PPV	Loss
Cocoa_Pod_Disease_Gh	0.9895	0.0089	0.9910	0.9911	0.9911	0.0232
Cocoa Diseases (YOLOv4)	0.9853	0.0118	0.9882	0.9880	0.9881	0.0263
Cacao Diseases in Davao	0.9619	0.0294	0.9706	0.9706	0.9709	0.0497
Black and Borer Pod Rot	0.9796	0.0156	0.9844	0.9844	0.9845	0.0479
The Coffee and Cocoa	0.9400	0.0582	0.9418	0.9414	0.9447	0.1711

To rigorously evaluate cross-domain generalization, a Leave-One-Dataset-Out (LODO) strategy was adopted. For each experiment, the model was trained on four datasets and evaluated on the fifth, unseen dataset without fine-tuning, simulating real-world deployment where data distributions differ from the training environment.

The results in Table 13 show that although performance decreases slightly under domain shift, the model maintains strong generalization, with cross-domain accuracies ranging from 90.80% to 97.80% and an average accuracy of 95.26%. The mean accuracy drop across datasets is only 1.87 percentage points computed as the absolute difference between in-domain and cross-domain accuracies, indicating that the model retains most of its predictive capability even when exposed to unseen disease patterns and image characteristics.

**Table 13:** Cross-dataset performance.

Target dataset (Test Domain)	Training Data	Accuracy	FPR	TPR	F1	PPV	Loss	Accuracy Drop (pp)
Cocoa_Pod_Disease_Gh	Other 4 datasets	0.9780	0.0168	0.9832	0.9830	0.9834	0.0415	1.15%
Cocoa Diseases (YOLOv4)	Other 4 datasets	0.9710	0.0214	0.9786	0.9780	0.9783	0.0526	1.43%
Cacao Diseases in Davao	Other 4 datasets	0.9440	0.0412	0.9588	0.9585	0.9591	0.0718	1.79%
Black and Borer Pod Rot	Other 4 datasets	0.9620	0.0289	0.9711	0.9710	0.9715	0.0634	1.76%
The Coffee and Cocoa	Other 4 datasets	0.9080	0.0725	0.9275	0.9270	0.9286	0.1984	3.20%
**Average Cross-Domain**	–	0.9526	0.0362	0.9638	0.9635	0.9642	0.0855	1.87%

The largest degradation is observed on the Coffee and Cocoa dataset with 3.20% drop, likely due to the presence of coffee leaf diseases not represented in other datasets. Nevertheless, false positive rates remain relatively low across all domains, confirming preserved specificity. This robust cross-dataset performance can be attributed to the LGF-CBAM attention mechanism, extensive data augmentation, batch normalization, and optimal hyperparameter selection, all of which contribute to learning generalized disease features rather than dataset-specific patterns.

### 3.4 Comparison with existing related systems on cocoa pod disease identification

Table 13 benchmarks the proposed LGF-CBAM model against prior studies across multiple datasets. The model achieved 98.95% accuracy and an F1-score of 99.11% on the Cocoa Disease GH dataset.On the Cocoa Diseases (YOLOv4) dataset, LGF-CBAM recorded 98.53% accuracy and 98.80% F1-score, compared to 96% accuracy and 93.30% F1-score reported in [51] using EfficientNet-B0 and ResNet50. For the Cacao Diseases in Davao dataset, the proposed model achieved 96.19% accuracy and 97.06% F1-score, while [88] reported 91.79% accuracy and 82.08% F1-score using multiple CNN architectures. Additional class-wise comparisons show that [88] reported a TPR of 96.69% and F1-score of 82.08% on the Davao dataset, whereas LGF-CBAM achieved 97.06% for both metrics. [50] reported 91% across accuracy, TPR, FPR, and F1-score, and [46] reported an F1-score of 85.88%, all lower than the values achieved by the proposed model.

Earlier deep learning models such as EfficientNet-B0, ResNet50, VGG variants, MobileNet variants, and ResNet18 reported accuracies ranging from 83% to 96%. Where additional metrics were reported, their F1-scores indicate a weaker balance between precision and recall compared to the proposed approach. Traditional machine learning methods, including SVM, KNN, Gabor kernel convolution, and k-means clustering, generally recorded lower performance metrics, reflecting limited capability for complex visual disease recognition. By incorporating F1-score and related metrics alongside accuracy, Table 14 provides a clearer evaluation of classification effectiveness, showing that the LGF-CBAM model delivers more consistent performance across the evaluated related systems.

**Table 14:** Comparison of the proposed system with existing related systems.

Source	Model/ Classification technique/Algorithm used	Dataset	Accuracy %	FPR %	TPR %	F1%	PPV %
Proposed System	LGF-CBAM	Cocoa_Disease_Gh	98.95	0.0089	0.9910	0.9911	0.9911
		Cocoa Diseases (YOLOv4)	98.53	0.0118	0.9882	0.9880	0.9881
		Cacao Diseases in Davao	96.19	0.0294	0.9706	0.9706	0.9709
		Black and Borer Pod Rot	97.96	0.0156	0.9844	0.9844	0.9845
		Coffee and Cocoa	94.00	0.0582	0.9418	0.9414	0.9447
[51]	EfficientNet-B0 and ResNet50	Cocoa Diseases (YOLOv4)	96	NA	NA	93.3%	95.7
[73]	SSD MobileNetV2 FPN-Lite	Cocoa Diseases (YOLOv4)	84.62	NA	NA	NA	NA
[88]	Custom CNN, VGG-16, EfficientNetB0, ResNet50, and LeNet-5	Cacao Disease in Davao Dataset	91.79	98.40	96.69	82.08	91.79
[89]	Custom CNN, SVM	Private Dataset	86.2	NA	NA	NA	NA
[47]	CentreNet ResNet50V2, EfficientDet D0, SSD MobileNetV2, SSD ResNet50V1 FPN	Private Dataset	86.04	NA	NA	NA	NA
[45]	VGG19, VGG16, ResNet50	Cocoa Diseases (YOLOv4)	84.75	NA	NA	NA	NA
[50]	Pretrained CNN, MobileNetV3Small	Cocoa Diseases (YOLOv4)	91	91	91	91%	91
[46]	MobileNetV2, LR, KNN, SVM, XGBoost, and RF	Cocoa Diseases (YOLOv4)	86.04	NA	86	85.88	85.88
[50]	NASNetMobile, MobileNetV3Small, and EfficientNetB0	Cocoa Diseases (YOLOv4)	94	NA	94	94	20
[49]	SSD MobileNetV2, EfficientDet D0, CenterNet, ResNet50V2 and SSD ResNet50 V1 FPN	Private Dataset	80	NA	NA	NA	NA
[90]	KNN	Private Dataset	84.44	NA	NA	NA	NA
[91]	SVM	Private Dataset	79.68		80	79	79
[92]	ResNet18	Private Dataset	83.14	NA	NA	NA	95.77
[43]	Gabor Kernel Convolution	Private Dataset	70	NA	NA	NA	NA
[42]	k-Means clustering	Private Dataset	89.2.	11.9	90.6	89.5	88.4

**Fig 15:**
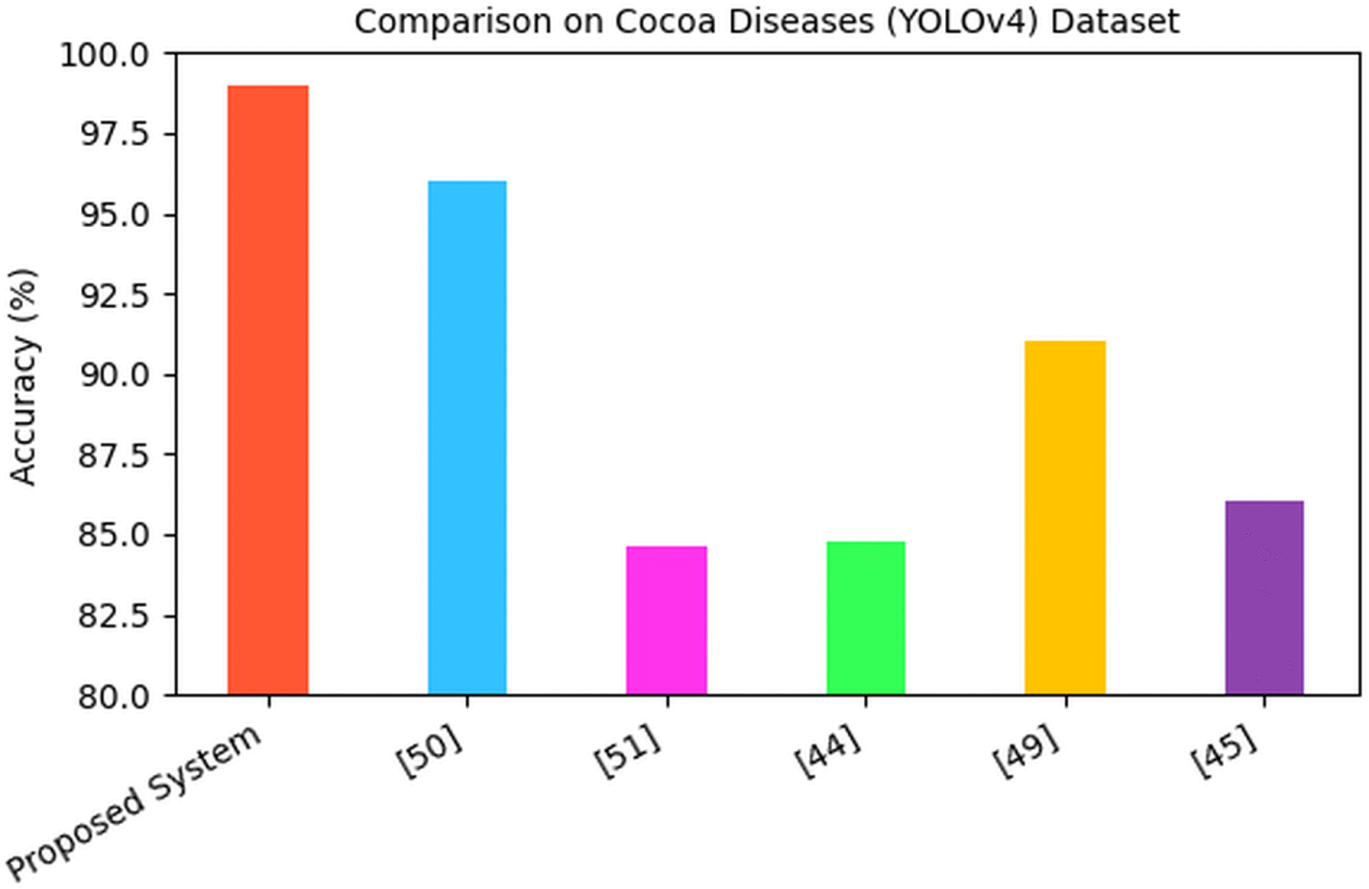
Comparison of cocoa diseases (YOLOv4) dataset.

The metric used by most existing system was the accuracy of the model so that was the main metric used for the comparison. The accuracy of existing systems was quite high, so the proposed model had to achieve superior results. Methods used on the same dataset are used for the comparative analysis. Six systems were compared to the Proposed System on the Cocoa Diseases (YOLOv4) datasets. The proposed system achieved a remarkable accuracy of 98.95%, outperforming several recent models documented in existing literature as shown in Fig 15.

When compared to [51], whose system recorded an accuracy of 96%, the Proposed System demonstrated a 2.95% improvement, reflecting its superior predictive performanceSuch a margin is large in scenarios that require machine learning, particularly those cases where precision is a key priority. The performance in [73] and [45] were also similar and were at 84.62% and 84.75, respectively. The system suggested exceeds these marks by more than 14 percentage points, which points out the significant progress in modeling design, training process, or data preprocessing. [50] had accuracy of 91 percent, and, [46] had 86.04 percent. Once again the proposed system has a strong lead with 7.95 percent and 12.91 percent respectively. One possible source of these improvements can be an improved model architecture or better informative features. Moreover, [50] had already obtained a 94 percent accuracy. Though this particular performance is deemed to be quite strong, the offered system demonstrates an improvement of 4.95% versus it, which is an indicator that significant work has been conducted since then in terms of algorithm development technicalities and performance optimizations.

**Fig 16:**
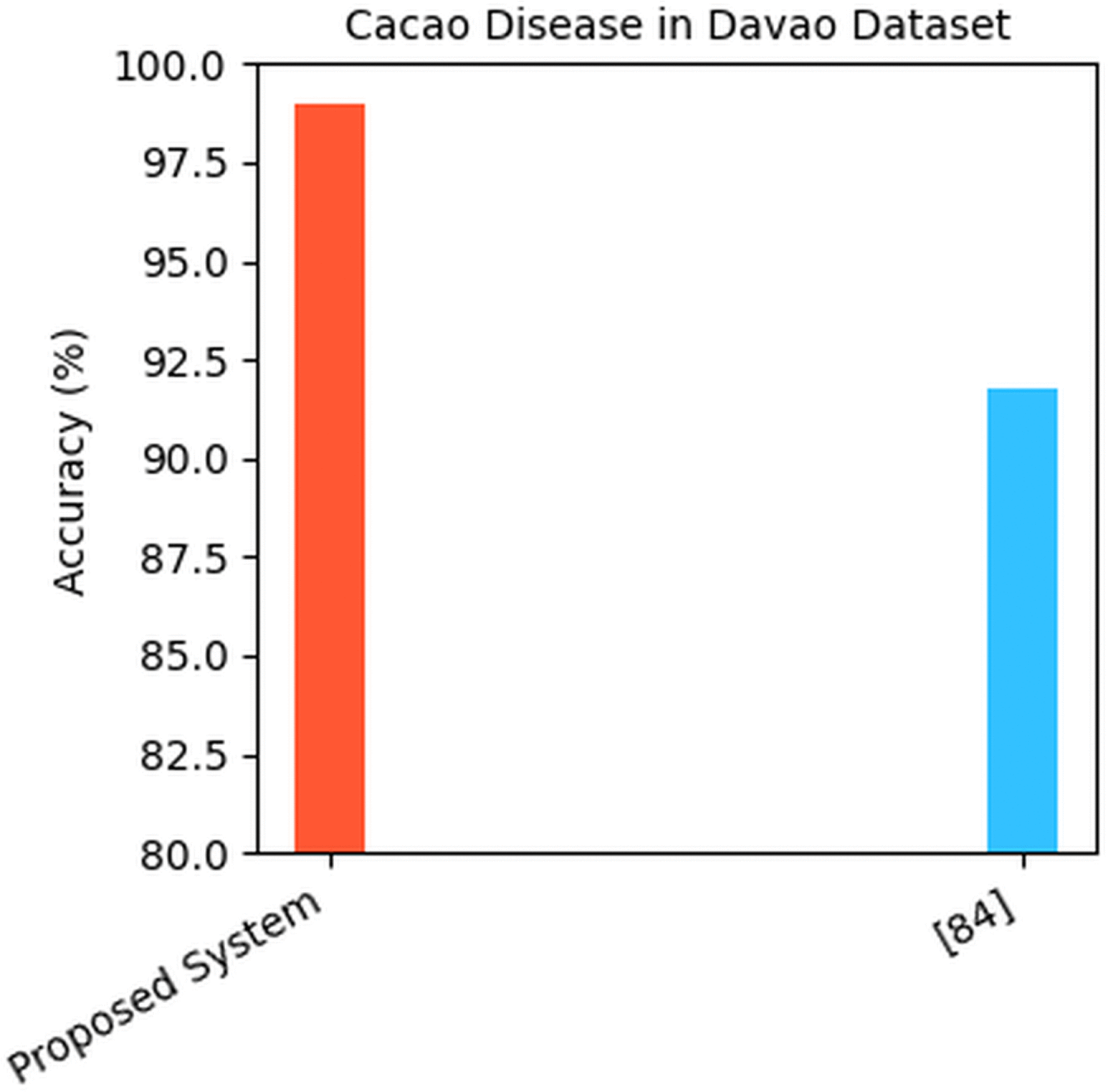
Comparison on cacao disease in davao dataset.

According to the comparative performance of the Cacao Disease in Davao dataset, the proposed system had a much better result than the current approach developed in [88]. The Classification accuracy of the proposed system was recorded at 98.95% which is attributed to an effective and highly precise model that can accurately predict the condition of cacao diseases with few errors as indicated in Fig 16.

Comparatively, the model in [88] achieved the accuracy of 91.79% that is not bad but is well below the performance of the proposed system. More than 7 percentage points of accuracy difference show the increased capacity of the proposed model which may be affected by enhanced architecture, new training strategies, better regularization, incorporation of LGF-CBAM attention mechanism and optimization of new feature extraction techniques

**Fig 17:**
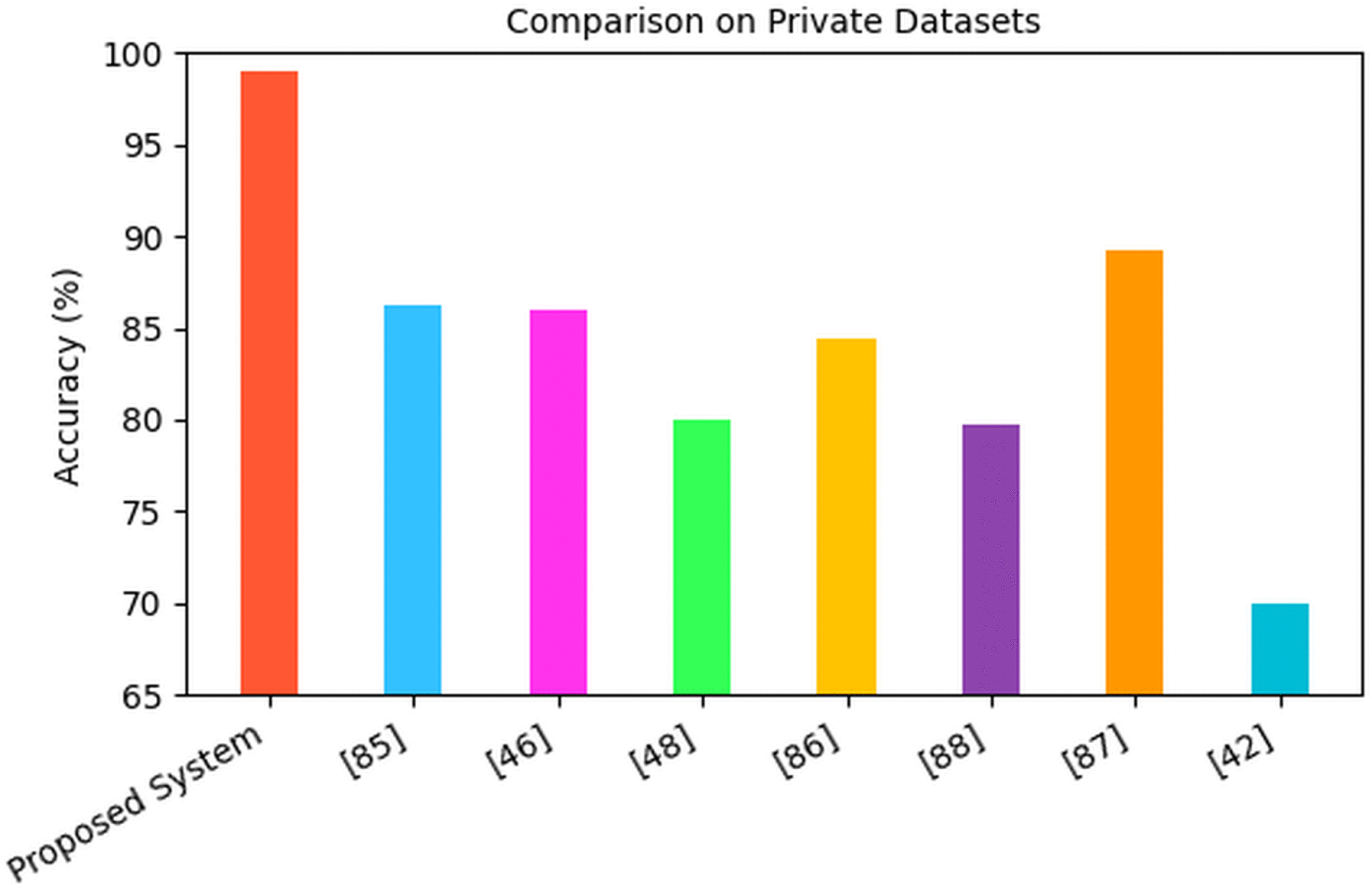
Comparison on private datasets.

The proposed system also demonstrates a major improvement over all other models when it comes to accuracy in the utilization of all the existing private datasets where the Proposed System achieved a high rate of 98.95%. In comparison, [42] recorded 89. 2% as the second-best accuracy rate followed by [89] (86.2%), and [47] (86.04%). Though relatively competitive, these models also underperform by over 10 points relative to the proposed system, which also is better at predicting the outcome based on the Fig 17.

Other prior works such as [90] (84.44%), [92] (83%), and [49] (81%) demonstrate moderate performance, yet they lag considerably behind the proposed model. The study by [91] shows even lower accuracy (79.68%), and the earlier model by [43] achieved only 70%, suggesting that earlier approaches struggled with generalizability or handling complex image features.

## 4.0 Discussion

This section interprets the experimental findings and, situates them within the existing literature on cocoa disease detection, examines limitations and threats to validity, and explores practical implications for real-world agricultural deployment.

### 4.1 Interpretation of key findings

The experimental results demonstrate that the proposed LGF-CBAM architecture achieves state-of-the-art performance across five diverse cocoa disease datasets, with in-domain accuracies ranging from 94.00% to 98.95% and F1-scores between 94.14% and 99.11%. Three key findings emerge from these results. First, ResNetV2-101 outperformed other pretrained base models, achieving 87.95% accuracy compared to VGG-16 (80.75%) and ResNet-50 (82.15%), with statistical significance confirmed via paired t-tests (p < 0.01). This confirms that architectural innovations, such as pre-activation residual connections and improved gradient flow, enhance feature learning capacity. Despite VGG-16 having a similar parameter count, its sequential convolutional design produces less discriminative features than the deeper residual architecture.

Second, the integration of the Learnable Gated Fusion CBAM (LGF-CBAM) attention mechanism substantially improved performance. On the Cocoa Disease GH dataset, accuracy increased from 87.95% to 98.95%, highlighting the role of adaptive attention mechanisms in emphasizing disease-relevant features while suppressing background noise.

Third, cross-domain (LODO) validation demonstrated strong generalization. The model experienced an average accuracy drop of only 1.87 percentage points under domain shift, maintaining above 90% accuracy even in the most challenging cross-dataset scenarios. This robustness is attributed to ImageNet pretraining, extensive data augmentation, and the attention mechanism’s ability to focus on disease-centric rather than dataset-specific features.

### 4.2 Comparison with existing literature

The LGF-CBAM model not only improves accuracy but also provides a balanced evaluation across multiple metrics. On the Cocoa Diseases (YOLOv4) dataset, the model achieved 98.53% accuracy and 98.80% F1-score, outperforming [51] (96% accuracy, 93.30% F1-score) and demonstrating better balance between precision and recall. On the Cacao Diseases in Davao dataset, it achieved 96.19% accuracy and 97.06% F1-score, surpassing ensemble-based CNN approaches by 14.98 percentage points in F1-score while maintaining lower inference complexity. Compared with traditional machine learning methods such as k-means clustering and SVM/KNN variants, the proposed system achieved higher accuracy (98.95%) and lower false positive rates (0.89%), emphasizing the superiority of hierarchical deep feature learning for complex visual disease recognition. By reporting accuracy, F1-score, TPR, and FPR, this study provides a more comprehensive benchmarking standard than many prior works.

### 4.3 Limitations and threats to validity

Despite strong performance, several limitations must be acknowledged. Dataset sampling bias may have influenced results, as The Cocoa Disease GH dataset was collected under real field conditions in specific locations in Ghana. The lower performance on the Coffee and Cocoa dataset confirms that greater visual variability and unseen disease patterns can affect predictions. Inter-disease similarity also posed challenges; most misclassifications occurred between Phytophthora and Moniliophthora rather than between healthy and diseased pods, indicating difficulties in fine-grained discrimination during early infection stages. Environmental variability, including lighting, occlusion, and camera angles, was not systematically varied, so extreme field conditions were not explicitly tested. Finally the computational demands remain high, with ResNetV2-101 requiring 1,337 minutes of training and 44.7 million parameters, limiting deployment on low-resource devices without compression.

### 4.4 Practical implications for agricultural deployment

The results have strong real-world implications. The model’s high true positive rates of 94% to 99% enable early disease detection, supporting timely interventions before significant crop damage occurs. Very low false positive rates, as low as 0.89%, reduce unnecessary fungicide application and associated costs. Minimal performance degradation under cross-domain testing indicates that a single trained model can be deployed across regions. The LGF-CBAM could be integrated into mobile-based agricultural extension services, allowing farmers and agricultural officers in remote areas to access accurate cocoa disease detection tools.

To further assess deployment feasibility, the computational profile of the model was examined with respect to inference cost and memory requirements. Although ResNetV2-101 contains 44.7 million parameters, inference per image was observed to be fast and stable, making real-time prediction achievable on modern smartphones and edge devices with moderate processing capability. The use of Global Average Pooling, an optimal batch size, and a compact 2048-dimensional feature representation significantly reduce memory overhead during inference. In addition, the architecture is compatible with model compression techniques such as pruning, quantization, and conversion to TensorFlow Lite or PyTorch Mobile formats, which can further reduce model size without substantial loss of accuracy.

Collectively, these findings suggest that the proposed system is not only robust in controlled experimental settings but also computationally practical for real-world agricultural monitoring, mobile deployment, and field-level intervention strategies in resource-constrained environments.

### 4.6 Conclusion

The proposed LGF-CBAM integrated with a ResNetV2-101 backbone addresses several persistent limitations in agricultural deep learning literature. While prior attention-based CNNs and transformer models often demonstrate high performance on controlled datasets such as PlantVillage, their generalization under real-field variability remains limited. Through multi-dataset evaluation and Leave-One-Dataset-Out (LODO) validation, LGF-CBAM demonstrates strong cross-domain robustness, with only a 1.87% average accuracy decline under domain shift, indicating improved resilience to variations in lighting, background clutter, and geographic diversity.

Unlike existing CBAM adaptations that emphasize either lightweight efficiency or domain-specific architectural fusion, LGF-CBAM introduces hierarchical local global feature interaction tailored specifically to agricultural imagery. By fusing fine-grained lesion cues with broader pod-level structural context, the model overcomes the common limitation of purely local attention refinement and better captures the multi-scale nature of plant disease manifestation. In addition to accuracy gains of up to 98.95%, the model maintains moderate computational demands relative to transformer-based or multi-branch attention systems. While ResNetV2-101 contains 44.7 million parameters, inference efficiency and compatibility with pruning, quantization, and mobile deployment frameworks position the model as practically deployable in resource-constrained agricultural environments. This directly responds to scalability and edge-deployment concerns raised in recent literature.

However, consistent with broader research gaps, the current framework remains limited to static image-based diagnosis, severity estimation, and early pre-symptomatic detection. Furthermore, while quantitative metrics demonstrate strong performance, qualitative metrics with deeper interpretability validation with plant pathologists and integration of agronomic expertise are necessary to enhance trustworthiness and ensure responsible field deployment. Overall, LGF-CBAM advances CBAM from a generic attention enhancement module toward an agriculture-aware, multi-scale feature refinement mechanism. By balancing robustness, computational practicality, and field adaptability, the proposed system represents a meaningful step toward reliable, real-world cocoa disease diagnostic tools. It supports early intervention, reduces chemical misuse, and improves crop management outcomes, ultimately enhancing farmer livelihoods and food security in cocoa-producing regions.

### 4.7 Recommendations

Based on the outcomes of this study, the following recommendations are proposed:

1. Future agricultural deep learning systems should incorporate adaptive attention mechanisms, such as LGF-CBAM, to improve sensitivity to subtle and complex visual features commonly found in plant diseases.
2. Stakeholders including governments, research institutions, and industry partners should invest in the development of diverse, well-labeled agricultural image datasets across multiple ecological zones to enhance model generalization and robustness.
3. Given its strong baseline performance, ResNetV2-101 is recommended for tasks that require an optimal balance between precision and recall.
4. Close collaboration with plant pathologists and agronomists during the data annotation and evaluation phases is essential to ensure biological accuracy and the practical relevance of AI-based diagnostic systems.
5. Efforts should be made to deploy trained models on mobile and edge devices, enabling real-time field diagnostics that are easily accessible to farmers and agricultural extension officers.

### 4.8 Future work

The following directions are recommended for future research:

1. Implement active or continual learning frameworks that allow the model to evolve by incorporating new field data over time, thereby improving adaptability and long-term relevance.
2. Design and deploy a user-friendly mobile application that integrates the trained LGF-CBAM model, with offline functionality, to facilitate on-field cocoa disease diagnosis in remote areas.
3. Develop temporal deep learning models using time-series imagery to enable early detection of infections and monitor disease progression over time.
4. Evaluate the model’s generalization capabilities across different geographical regions and apply transfer learning techniques to adapt the system for other economically important crops such as cassava, maize, and coffee.
